# Is Caloric Restriction Associated with Better Healthy Aging Outcomes? A Systematic Review and Meta-Analysis of Randomized Controlled Trials

**DOI:** 10.3390/nu12082290

**Published:** 2020-07-30

**Authors:** Silvia Caristia, Marta De Vito, Andrea Sarro, Alessio Leone, Alessandro Pecere, Angelica Zibetti, Nicoletta Filigheddu, Patrizia Zeppegno, Flavia Prodam, Fabrizio Faggiano, Paolo Marzullo

**Affiliations:** 1 Department of Translational Medicine (DIMET), Università del Piemonte Orientale, 28100 Novara, Italy; silvia.caristia@uniupo.it (S.C.); marta.devito@uniupo.it (M.D.V.); andrea.sarro@uniupo.it (A.S.); alessio.leone1991@gmail.com (A.L.); alessandro.pecere@uniupo.it (A.P.); angelica.zibetti@uniupo.it (A.Z.); nicoletta.filigheddu@uniupo.it (N.F.); patrizia.zeppegno@uniupo.it (P.Z.); fabrizio.faggiano@uniupo.it (F.F.); 2 Department of Health Sciences (DISS), Università del Piemonte Orientale, 28100 Novara, Italy; flavia.prodam@uniupo.it; 3 IRCCS Istituto Auxologico Italiano, Ospedale S. Giuseppe, 28824 Piancavallo, Italy

**Keywords:** randomized controlled trials, caloric restriction, predictors, hormones, cardiovascular risk, psychological wellbeing, healthy aging, longevity

## Abstract

Background: Global dietary patterns have gradually shifted toward a ‘western type’ with progressive increases in rates of metabolic imbalance. Recently, animal and human studies have revealed positive effects of caloric restriction (CR) on many health domains, giving new knowledge for prevention of ill and health promotion; Methods: We conducted a systematic review (SR) of randomized controlled trials (RCTs) investigating the role of CR on health status in adults. A meta-analysis was performed on anthropometric, cardiovascular and metabolic outcomes; Results: A total of 29 articles were retrieved including data from eight RCTs. All included RCTs were at low risk for performance bias related to objective outcomes. Collectively, articles included 704 subjects. Among the 334 subjects subjected to CR, the compliance with the intervention appeared generally high. Meta-analyses proved benefit of CR on reduction of body weight, BMI, fat mass, total cholesterol, while a minor impact was shown for LDL, fasting glucose and insulin levels. No effect emerged for HDL and blood pressure after CR. Data were insufficient for other hormone variables in relation to meta-analysis of CR effects; Conclusion: CR is a nutritional pattern linked to improved cardiometabolic status. However, evidence is limited on the multidimensional aspects of health and requires more studies of high quality to identify the precise impact of CR on health status and longevity.

## 1. Introduction

Interest in determinants of lifelong health has progressively grown, along with the search of molecular pathways to longevity. Diet—in particular, caloric restriction (CR)—seems to play a crucial role on longevity via processes relating to metabolic adaptation and reduced lipogenesis, so as to draw the attention of recent scientific research on potential therapeutic implications [1,2,3,4,5,6]. CR—a non-pharmacological dietary procedure increasingly associated with protection against oxidative stress and metabolic disease—is a nutritional pattern based on the reduction of the average daily caloric intake, without generating malnutrition or deprivation of essential nutrients.

A large number of animal studies demonstrated a strong correlation between CR and the increase in lifespan or the reduction in age-related chronic disease rates. Animals kept on a CR diet showed a significant increase in life expectancy by approximately 50% [7,8]. In animal studies, Li et al. reported on the favorable effect of short-term CR on cardiometabolic alterations, while Yzydorczyk et al. showed that short-term CR can reverse hepatic alterations and notably cellular senescence [9,10]. In humans, experimental and observational investigations are increasingly revealing that CR is able to reduce the incidence of cardiovascular diseases, diabetes, dementia, frailty and cancer [11,12].

In order to better understand the role of CR on molecular mechanisms underlying its health benefits, several hypotheses and theories have been developed, including those dealing with the importance of regulatory proteins such as mTOR, sirtuins and IGF-I, which regulate cells growth, proliferation and endurance [13,14]. Over the last few years, the increasing interest regarding CR led to conduct several randomized controlled trials (RCTs) aimed to assess the effects and the impact of this dietary pattern on humans in relation to markers of health status [15,16,17,18].

Uncertainty remains about the risk/benefit balance of CR and its transferability to the current medical practice. Although during the last years different reviews and SRs were published related effect of some type of CR on health, there are still no systematic reviews (SRs) quantitatively summarizing the potential association between CR and multiple dimensions of health status. In fact, different SRs have explored the association between CR and asthma [19], hypercholesterolemia [20], cardiovascular health [4] or bone health [21]. On the other hand, some SRs have examined the general effects of diet [22] or intermittent energy restriction [23] on health, while others took in consideration specific populations such as intensive care units patients [24], athletes [25] or animal models [26]. Finally, Miller and colleagues [27] analyzed the effect of CR in combination with physical exercise, whereas studies published by Seyfried et al. [28], Omodei et al. [29] and Locher et al. [30] did not represent systematic reviews of current evidence on CR.

Hence, the aim of this study was to assess the effects of CR on dimensions of the WHO health concept [31,32], with a systematic review and meta-analyses of RCTs performed on this topic.

## 2. Methods

We conducted a SR of randomized controlled trials (RCTs) on the effects of CR in humans. To achieve this goal, the study protocol was organized according to PRISMA-P guidelines [33], and the resulting report was written according to the Preferred Reporting Items for Systematic Reviews and Meta-Analyses (PRISMA) [34,35]. The protocol was not registered.

### 2.1. Eligibility Criteria

We selected RCTs conducted for the purpose of investigating the relation between CR and health dimensions. Studies in this SR were selected according to the following criteria of eligibility:

- Population: studies on people aged ≥18 years;
- Intervention: CR with a reduction of daily energy caloric intake equivalent to 20–30% of energy requirements, expressed as kcal/day;
- Controls: any type of control population not exposed to CR;
- Outcomes: biomarkers of health status, risk factors for illness, mortality [36,37], quality of life and well-being;
- Study design: RCT;
- Setting: any type of setting;
- Period: papers published before 31 March 2019;
- Language: English.

### 2.2. Search Strategy

For the purpose of our SR, we searched Medline (PubMed) and ClinicalTrials.gov (www.ClinicalTrials.gov). Databases were searched independently by two reviewers (SC and MDV), and the opinion to third reviewer was required in case of disagreement.

We used a string including Mesh terms: “caloric restriction, aging and humans”, combined with the following key-words: “low calorie diet, lipid restriction, fat reduction, antiaging, healthy aging, mortality, survival, metabolic diseases, diabetes, Alzheimer’s”.

PubMed and ClinicalTrials.gov were consulted on 31 March 2019. Search strategies are detailed in Table S1 search strategy.

Potentially relevant literature and other eligible studies were further identified using a snowballing approach by screening the reference lists of all previously identified studies [38].

Studies identified in Clinicaltrials.gov were then searched in Medline in order to assess publication bias.

### 2.3. Study Selection and Data Extraction

Once the records retrieved by the search were deduplicated, two authors (MDV and AL) independently reviewed the titles and the abstracts in order to identify studies to be included in the review. Any disagreement was discussed with another reviewer (SC). Finally, the full texts of articles passed the screening and eligibility steps were retrieved and read by the same reviewers. Disagreements were documented and resolved after discussion, in the wider team when needed.

Two reviewers independently extracted data from the included trials (MDV and AL). They collected mean values and their margins of error (as ± SD or 95% CI) at baseline and follow-up. If the selected RCTs reported more than one follow-up measurement, we collected those being closer to 12-months from baseline.

### 2.4. Risk of Bias in Individual Studies

The methodological quality of the included studies was assessed using the Cochrane’s risk of bias tool [39]. Two reviewers independently evaluated the following risk of bias (SC and MDV) criteria for each of the included papers: random sequence generation, allocation concealment, presence of blinding (participants, staff and outcome assessors) in the studies, incomplete outcome data and selective outcome reporting. Risks of bias were assessed as recommended: low risk, high risk or unclear risk. Disagreements were resolved by discussion between the reviewers.

### 2.5. Outcomes

Primary outcomes were related to anthropometric measures: weight, BMI and fat mass; cardiovascular risk factors: systolic (SBP) and diastolic blood pressure (DBP), total cholesterol, LDL cholesterol, HDL cholesterol, triglycerides, free fatty acids; hormone and metabolic biomarkers: IGF-1, IGFBP-1 and -3, leptin, T3, T4, ghrelin, adiponectin, GH, fasting insulin and glucose, insulin sensitivity; bone: C-terminal telopeptide of type 1 collagen, tartrate-resistant acid phosphatase isoform, serum sclerostin, bone mineral density; inflammatory and oxidative stress markers: white blood cell count, C-reactive protein (CRP), TNF-alfa, interleukins, mitochondrial DNA, nitric oxide-related signaling markers).

Secondary outcomes identified indicators and scales used for well-being and quality of life measurement: Cantril’s ladder of life scale [39], scale of positive and negative experience (SPANE) [40], the European social survey well-being [41], the life satisfaction scale/life satisfaction index (LSI) [42,43], the CES-D [44], psychological well-being scale (PWB) [40], the flourishing index [45] or the geriatric depression scale [46]).

### 2.6. Summary Measures and Synthesis of Results

A meta-analysis was performed on measures presented as the results in at least three studies. According to the current search, these measures included: bodyweight, BMI, fat mass, systolic and diastolic blood pressure, HDL, LDL, total cholesterol, fasting insulin and fasting glucose levels. The remaining outcomes were narratively synthetized.

Meta-analyses were conducted using a random effects model by DerSimonian and Laird’s method [47] and calculating mean difference (MD) and 95% CI of outcomes at follow-up (and/or mean change from baseline to follow-up), expressed as the same measure unit (e.g., bodyweight, BMI, fat mass, HDL, LDL, total cholesterol, blood pressure and fasting glucose).

If articles included baseline and follow-up values, but unbalance was recorded at baseline, especially for cardiovascular and metabolic risk factors, then inter-group mean changes of variables and their ±SD were calculated following Cochrane’s method and SD values were assigned to changes from baseline when values of changes were missing [48]. In the presence of different measure units, data were converted to the same unit.

Statistical heterogeneity was explored and analyzed quantitatively by I-squared (I^2^) test, which was judged acceptable if ≤30%; in case of higher I^2^ results, heterogeneity was further investigated using meta-analysis by subgroups (follow-up lengths, BMI, health status and other risk factors at baseline) [49].

In spite of randomization, some studies showed unbalanced groups at baseline for some outcomes (e.g., blood pressure, lipid profile, insulin and glucose). This caveat was attributed to simple randomization process in the presence of a small sample size [50] or to stratification for specific variables. For example, some RCTs randomized for sex and/or BMI, but without considering other variables such as blood pressure, lipids, insulin and glucose profile. In such cases, due to the lack of data sufficient for ANCOVA in full texts, sensitivity analyses were conducted by separating meta-analyses as balanced and unbalanced at baseline for these outcomes if necessary.

Finally, we tested the risk of bias across studies using the Egger’s test for small-studies effect investigation and funnel plots were used to examine publication bias [51,52].

## 3. Results

The initial PubMed search retrieved 182 records (search No. 1 in Table S1 search strategy), while 262 additional titles were found in ClinicalTrial.gov. Cumulatively, our search collected 444 records, while 481 further records were identified through a snowball method from references of papers identified through PubMed. After record deduplication (833 residual records) and selection by title, the remaining 344 records were screened by reading abstracts, and 213 were excluded for several reasons. Out of these 213, 49 RCTs had not yet recruitment any participants and other 49 articles did not report data about RCT studies (animals/population criteria *n* = 52, not yet recruitment participants *n* = 49, RCTs criteria *n* = 38, caloric restriction topic *n* = 34, multiple reasons *n* = 31, intervention criteria *n* = 4, results lacking *n* = 4 and comparison group criteria *n* = 1). One hundred and thirty-one articles were identified by at least one assessor as potentially eligible, and their full text was obtained on the basis of our predefined eligibility criteria. Among these, 102 records were excluded for reasons relating to the study design (*n* = 25) or because involving animal studies (*n* = 13) or due to other reasons (*n* = 64).

Out of 12 eligible studies registered as protocols in ClinicalTrial.gov, 9 RCTs resulted to be started, but had not been completed, while 3 further studies could not be retrieved as publications. All had been deposited in the ClinicalTrial.gov registry for at least 5 years before our analysis was conducted.

**Figure 1:**
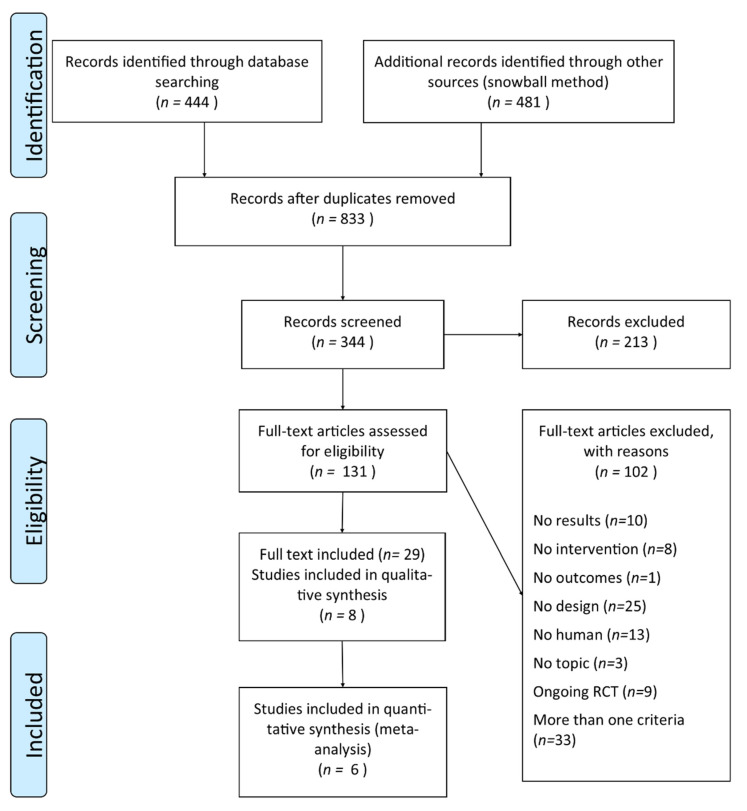
Preferred Reporting Items for Systematic Reviews and Meta-Analyses (PRISMA) flow diagram of the study.

We included in this SR 29 articles found from search strategy, as previously presented. These are publications of results regarding 8 RCTs (included in this SR). Out of these 8 studies included, 6 studies entered in analyses for quantitative summarizing of results (meta-analysis). The selection process is summarized in Figure 1.

### 3.1. Study Characteristics

This SR included 29 articles, reporting data from 8 RCTs (Table 1). To link our analyses to study cohorts rather than results of individual publications, the first author and publication years for each study were quoted throughout the main text. A full list of articles herein included is detailed in Table S2 List of Included Studies.

Articles included in this SR collectively encompassed 704 subjects, 64% of whom were women. With regards to the country of origin, 7 studies had been carried out in the United States and one in Malaysia [53]. The age of patients included in the trials at baseline ranged between 31–70 years. Five studies recruited only healthy people. Of the remainders, one study recruited people with hypertension [53], another recruited older obese subjects with mild-to-moderate frailty [54] and the last recruited subjects with obesity-related comorbidities [16]. Overall, 82% of the subjects included in this SR were categorized as healthy, while 50% were classified as obese or overweight. Further information is reported in Table S3 Table of results.

Among the 334 subjects who completed the CR trials, the compliance to intervention appeared to be generally acceptable (Table S3 Table of results): four studies reported a “high” or “good” compliance or adherence to diet (Armamento-Villareal 2012, Buchowski 2012, Heilbronn 2006, Sparks 2016), whereas one study did not report on compliance [16]. Buchowski 2012 monitored compliance to the intervention through urinary biomarkers, such as ratios of protein, sodium and potassium intake/excretion [15]; Armamento-Villareal 2012 and Heilbronn 2006 monitored adherence to diet with self-reported records (e.g., food diaries) and changes in body weight [55,56]; finally, Sparks 2016 monitored adherence by assessing weight loss equivalent at least to 5% of initial body weight during the first year [57]. None of them specified the size of adherence in terms of people compliance with intervention or% of caloric reduction. In 2 studies, subjects’ compliance was higher during the first 6 months and declined thereafter, and the calculated reduction in daily energy intake approximated 10% [58,59].

### 3.2. Risk of Bias within Studies

The risk of bias assessment for clinical trials are provided in Table 2 [60]. All trials were at risk of bias for at least 1 of the assessed domains. However, 7 RCTs (87.5%) adequately generated their randomization sequence and five (55.5%) adequately concealed allocation. Oppositely, 3 studies (37.5%) showed a high risk for attrition bias and five (62.5%) for reporting bias. All RCTs included in our SR were at low risk for performance bias related to objective outcomes.

### 3.3. Findings

Studies included in our SR reported effects of CR on anthropometric measures, bone health, cardiovascular risk factors, hormonal and metabolic homeostasis and mood as detailed in the primary and secondary outcomes sections. Data reported by at least 3 studies have been quantified by meta-analysis, while the remainders have been described narratively (Table S3 Table of results).

#### 3.3.1. Anthropometric Measures

The 8 studies herein included have reported data on anthropometric and body composition outcomes (Table 2). On average, meta-analysis showed that CR determined a loss of 7.9 kg in bodyweight (95% CI −7.99; −7.81), 2.68 kg/m^2^ in BMI (95% CI −3.51; −1.86) and 4.40 kg in fat mass (95% CI −6.69; −0.45) (Table 3). Weight loss was greater in studies lasting longer than 6 months than those with shorter follow-ups and among overweight than obese subjects (Table 3) [15,18,57,59]. Additionally, Heilbronn 2006 reported significant reductions in visceral (−28% ± 4%; *p* < 0.005) and subcutaneous abdominal adipose tissue (−26% ± 4%, *p* < 0.005) in CR-exposed participants [62].

#### 3.3.2. Cardiovascular Risk Factors

##### Arterial Blood Pressure

**Figure 2:**
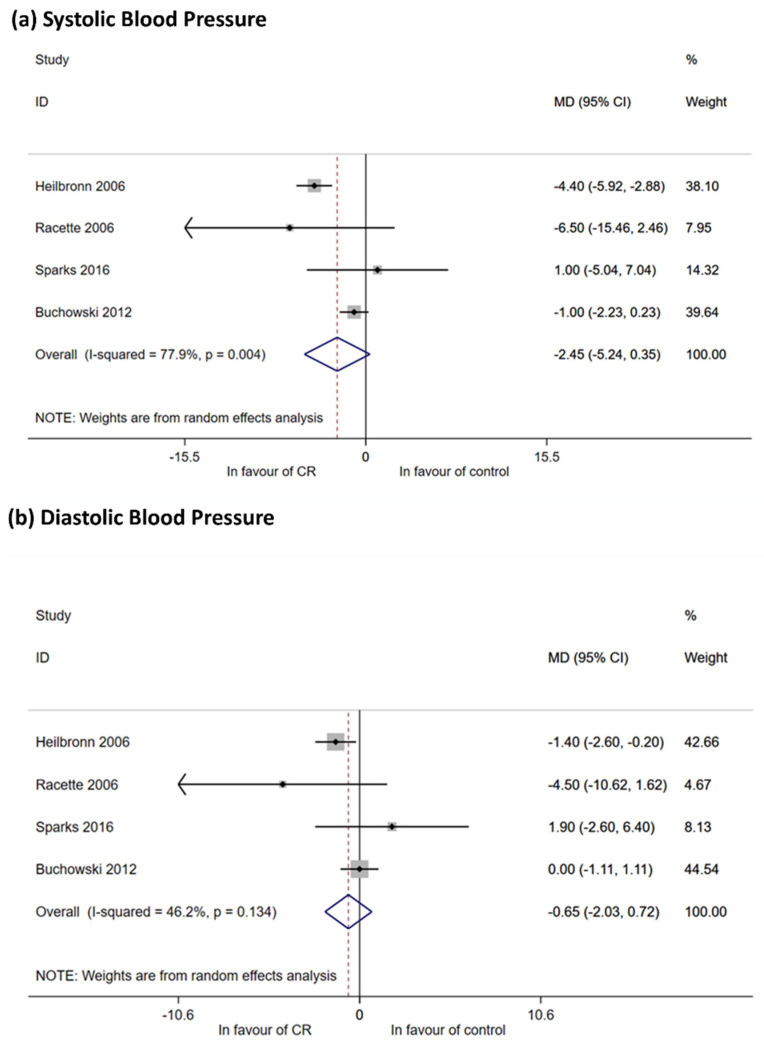
Meta-analysis for systolic (SBP) and diastolic blood pressure (DBP). (**a**) Meta-analysis using random effect method, outcome SBP. Meta-analysis was performed using post mean value (or median value) or mean change within groups and calculating mean differences (with their 95% CI). Four RCTs reported sufficient data for quantitative synthesis show a reduction of SBP among intervention participants, but 95% CI includes non-difference value (zero). Heterogeneity is high. All mean differences (MD) are expressed in mmHg; (**b**) meta-analysis using random effect method, outcome DBP. Meta-analysis was performed using post mean value (or median value) or mean change within groups and calculating mean differences (with their 95% CI). Meta-analysis with all four studies included shows none effect on DBP reduction. Heterogeneity is moderate. All MDs are expressed in mmHg.

Four studies (Heilbronn 2006, Racette 2006, Buchowski 2012, Sparks 2016) [15,18,57,63] recruited subjects without hypertension at baseline and reported outcomes on blood pressure (Table S3 Table of results). Meta-analysis showed a reduction in SBP (−2.45 mmHg, 95% CI −5.24; 0.35) and DBP (−0.65 mmHg, 95% CI −2.03; 0.72) that did not reach significance (Figure 2a,b). One study (Buchowski 2012) failed to document variations in DBP [15]. In sensitivity analyses, the effect of CR was only observed in unbalanced RCTs at baseline (Table 2 in Table S3 Table of results and Figure S1 Forest and Funnel Plots).

##### Lipids

**Figure 3:**
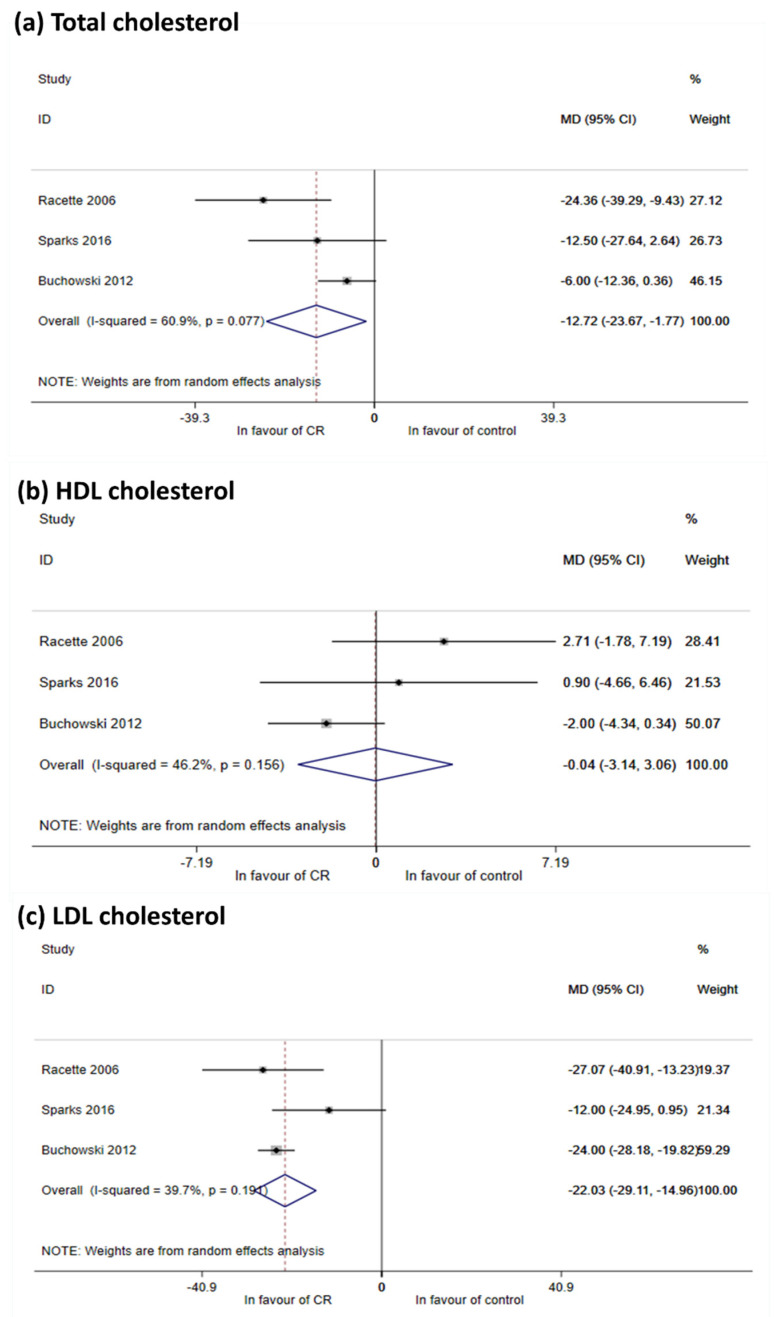
Meta-analysis for LDL, HDL and total cholesterol. (**a**) Meta-analysis using random effect method, outcome Total cholesterol. Meta-analysis was performed using post mean value (or median value) or mean change within groups and calculating mean differences (MD, with their 95% CI). Despite the moderate heterogeneity, meta-analysis shows that CR is effective in total cholesterol reduction All MDs are expressed in mg/dL; (**b**) meta-analysis using random effect method, outcome HDL cholesterol. Meta-analysis was performed using post mean value (or median value) or mean change within groups and calculating mean differences (with their 95% CI). Mean estimate is in favor of control, but non statistically significant. Only Racette 2006 showed an iatrogenic effect of CR. All MDs are expressed in mg/dL; (**c**) meta-analysis using random effect method, outcome LDL cholesterol. Meta-analysis was performed using post mean value (or median value) or mean change within groups and calculating mean differences (with their 95% CI). Impact on LDL reduction is showed considering all studies together. Heterogeneity is moderate. All MDs are expressed in mg/dL.

Three studies reported findings on lipids (Racette 2006, Buchowski 2012, Sparks 2016) [15,58,64]. Meta-analysis documented a reduction in total cholesterol (−12.72 mg/dL, 95% CI −23.77; −1.77) and in serum LDL levels (−22.03 mg/dL, 95% CI −29.11; −14.96) (Figure 3a–c). Effects appeared somewhat milder but were still significant in RCTs that were balanced at baseline (Table 2 in Table S3 Table of results and Figure S1 Forest and funnel plots). Although no effects were seen for HDL cholesterol (Figure 3), Heilbronn 2006 observed an increase of HDL at 6-months follow-up (Table S3 Table of results). Following CR, these authors also observed a reduction in intrahepatic lipid (−37% ± 10%, *p* < 0.01 from baseline), but not in intramyocellular lipids [17]. Finally, Ravussin 2015 reported an effect of CR on circulating triglycerides [59], while Racette 2006 reported a reduction of free fatty acid levels after intervention, but this change was not different from that seen in controls [64]. Oppositely, Heilbronn 2006 [62] showed an increase in free fatty acid levels.

#### 3.3.3. Hormonal and Metabolic Homeostasis

##### Hormone Profiles

Heilbronn 2006 [65], Racette 2006 [66] and Ravussin 2015 [67] analyzed the association between CR regimen and IGF-1 and IGFBP-3 concentrations, without relevant findings. In addition, Ravussin 2015 [67] assessed IGFBP-1 documenting a 25% increase compared to baseline, which was reflected in a 42% decrease of the IGF-1/IGFBP-1 ratio compared to the control group. Reductions in leptin concentrations were reported in groups exposed to CR by Heilbronn 2006 [68], Buchowski 2012 [15] and Ravussin 2015 [69,70]. Expectedly, leptin changes were related to reductions in fat mass [68]. A significant decrease in T3 concentrations was documented following CR by Racette 2006 [71], Ravussin 2015 [59] and Heilbronn 2012 [18] (−9.8 ± 2.0 ng/dL, *p* < 0.001; −18.4 ± 1.8 ng/dL, *p* < 0.001; −8.9 ng/dL, *p* < 0.02;, respectively). In addition, T4 levels were measured by Heilbronn 2006, who showed a decrease of this hormone. A single study examined the association between the variation in thyroid hormone and leptin (r^2^ = 0.22, *p* = 0.01) [59]. Finally, an incremental response was noticed after CR for ghrelin (+7% ± 1%; *p* = 0.03) [59] and adiponectin (+2.2 ± 4.7 µg/mL; *p* = 0.005) [64], while no change in GH was recorded [59] (Table S3 Table of results).

##### Glucose Metabolism

**Figure 4:**
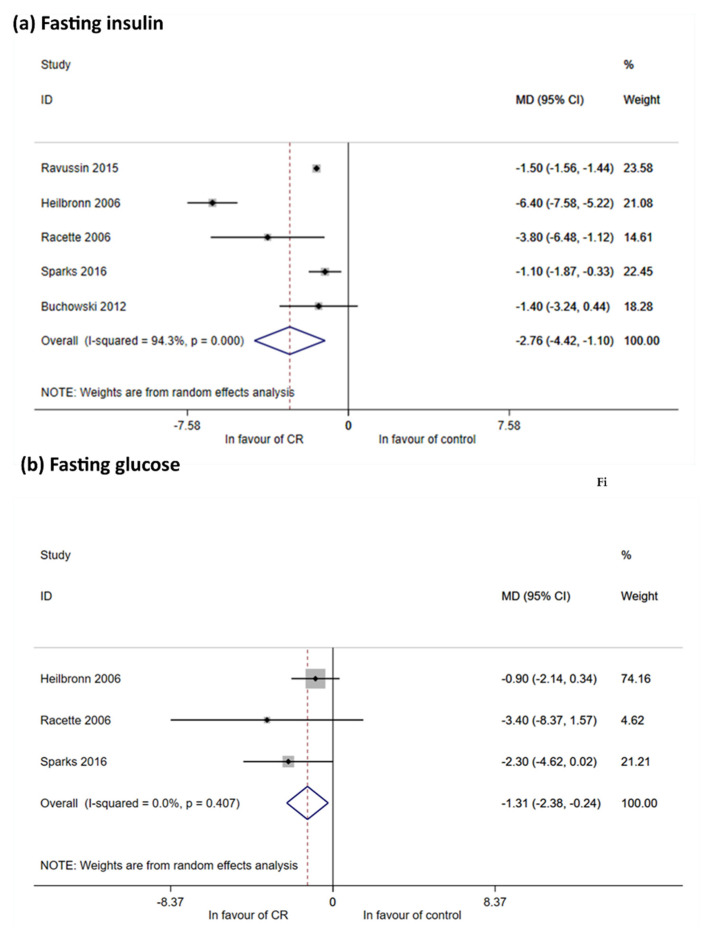
Meta-analysis for fasting glucose and insulin. (**a**) Meta-analysis using random effect method, outcome fasting insulin. Meta-analysis was performed using post mean value (or median value) and calculating mean differences (with their 95% CI). Impact on fasting insulin reduction is showed considering all studies together. I^2^ is high. All estimates are expressed in mIU/L; (**b**) meta-analysis using random effect method, outcome fasting glucose. Meta-analysis was performed using post mean value (or median value) or mean change within groups and calculating mean differences (with their 95% CI). Impact on fasting glucose reduction is showed by overall estimate with null heterogeneity. All MDs are expressed in mg/dL.

Five RCTs reported data on fasting insulin: Heilbronn 2006 [18], Racette 2006 [64], Buchowski 2012 [15], Ravussin 2015 [69] and Sparks 2016 [57]. All showed a reduction of fasting insulin after CR in comparison to controls (MD, −2.76 mIU/L; 95% CI, −4.42 to −1.10 mIU/L) (Figure 4a). However, in sensitivity analysis (Table 2 in Table S3 Table of results) no statistical significance was reached due to the presence of unbalanced groups (Racette 2006 and Sparks 2016). Data on fasting glucose were reported by Heilbronn 2006, Racette 2006 and Sparks 2016 [17,57,64] and all were used for meta-analysis (Figure 4b). A reduction was seen in fasting glucose by −1.31 mg/dL (95% CI −2.38 to 0.24 mg/dL), with an effect only for balanced group (−1.24 mg/dL 95% CI −2.42 to −0.06 mg/dL) (Table 2 in Table S3 Table of results and Figure S1 Forest and funnel plots).

Finally, two studies, Racette 2006 [66] and Heilbronn 2006 [62,68,72], showed an improvement in insulin sensitivity that was associated with reductions in whole-body fat mass (r = −0.46; *p* = 0.001), visceral adipose tissue (r = −0.51; *p* < 0.01) and subcutaneous abdominal adipose tissue (r = −0.32; *p* < 0.05) [62,68,72].

#### 3.3.4. Bone Health and Osteoporosis

Three studies [15,18,57] reported results on bone health, but outcomes were heterogeneous and data resulted insufficient for metanalysis. Overall, findings for bone health and risk of osteoporosis were discordant. Ravussin 2015 [59] reported an increase after 6 months of bone resorption markers, such as the C-terminal telopeptide of type 1 collagen (0.098 ± 0.12 µg/L; *p* < 0.001) [69] and tartrate-resistant acid phosphatase isoform (0.4 ± 0.1 U/L; *p* = 0.004) with a barely significant reduction, at 24 months, of bone formation predictors (e.g., bone-specific alkaline phosphatase: −1.5 ± 0.4 U/L; *p* = 0.047). In the study by Armento-Villareal 2012, the CR group manifested a progressive increase (10.5% ± 1.9%; *p* < 0.05) of serum sclerostin levels, a bone resorption marker associated with cortical thickness and changes in bone mineral density assessed at the femoral neck [55]. While Heilbronn 2006 did not reveal any change in bone structure at the 6 month follow-up [73], Ravussin 2015 found a reduction in bone mineral density at the lumbar spine, hip and femoral neck, trochanter and forearm after 24 months from baseline in the CR group [69].

#### 3.3.5. Inflammation and Oxidative Stress

Five studies [15,18,58,59,60] reported findings on inflammatory markers, but data were scattered and results were insufficient to generate a meta-analysis. Ravussin 2015 described a significant reduction in white blood cell count, particularly lymphocytes (−0.207 × 10^3^/µL; *p* < 0.0001), as well as in CRP levels [70]. Two RCTs analyzed serum TNF-alfa concentrations and either reported no change [64] or observed a reduction by 50% (*p* = 0.025) [70] following CR. While no changes were documented by Ravussin 2015 in other inflammation biomarkers, e.g., IL-6, IL-8 and IL-1beta, [70], Buchowski 2012 showed differences of IL-8 and IL-12 in CR group in comparison to control. Both IL-8 and IL-12 were lower in CR subjects than controls after 1 month, while only IL-12 remained statistically lower than controls after 4 months [15]. Heilbronn 2006 [74] and Sparks 2016 [57] evaluated oxidative processes, mitochondrial function and associated transcriptional profiles in CR group compared to no-intervention groups, with opposite results. While Heilbronn 2006 [74] found substantial effects of CR on mitochondrial biogenesis with a significant increase of mtDNA, as well as genes related to nitric oxide signaling and mitochondrial functions, Sparks 2016 [57] revealed that a small subset of genes related to nitric oxide signaling, particularly phosphodiesterases and mtDNA copy number were significantly downregulated after CR. These RCTs observed a reduction in oxidative DNA damage [57,74].

#### 3.3.6. Secondary Outcomes: Mood Disorders, Well-Being and Quality of Life

Data from available studies on psychological traits were insufficient for meta-analysis. Ravussin 2015 reported a significant reduction in the likelihood of experiencing mood disorders after CR (MD, −0.76; 95% CI, −1.41 to −0.11) [75]. Two studies, Teng 2011 [53] and Ravussin 2015 [75] analyzed persons’ well-being and quality of life. Ravussin 2015 observed an increase in scale scores in favor of CR, reporting a MD of 6.45 (95% CI 3.93–8.98; *p* < 0.05) [75]; Teng 2011 reported in the intervention group an 8.3% score increase from baseline, whereas the increment was less evident in the control group (6.2%; *p* <0.05) [53].

In relation to subscales of QoL, Ravussin 2015 reported a slight decrease in stress after CR in comparison to controls (MD, −0.79; 95% CI, −1.38 to −0.19) and a marginal improve of sleep perception (MD, −0.26; 95% CI, −0.49 to −0.02) [75]. Teng 2011 did not show modifications in the perception of stress and sleep quality, but they noticed significant increments in vitality in the CR group with respect to controls (+8.7% vs. +5.9%; *p* <0.05) [53].

### 3.4. Bias Across Studies

Egger’s test did not reveal statistically significant bias for small-study effects. However, funnel plots in Figure S1 Forest and Funnel plots were asymmetric toward the left side of the plot (except for HDL whose dots are mainly distributed on the right) reflecting a possible publication bias in favor of CR.

## 4. Discussion

Caloric restriction is a recognized non-genetic modulator of health-span in animal and human models. Cutting up daily nutrient intake triggers biochemical and molecular mechanisms that contribute to halt cardiometabolic disorders and other age-associated diseases [76]. In Rhesus monkeys, beneficial effects of CR have been described for immune, neuro–motor and sarcopenia outcomes [8]. In humans, CR feasibility, safety and effects on energy homeostasis and anti-inflammatory predictors of longevity have been underscored [59]. The molecular mechanisms underlying this multifold effect in rodent species include a lower oxidative damage and mitochondrial free radical generation [77]. In mammals, molecular pathways linked to CR include sirtuins and mTOR pathways, particularly Sirt1 and its ability to promote lipolysis through PPARγ inhibition [78]. Moreover, metabolic regulatory effects of CR include attenuation of protein translation through inhibition of mTORC1 and improved glucose homeostasis through inhibition of mTORC2 [79]. However, the consistency and reproducibility of results remain variable across different investigations and key questions remain unanswered on timing, duration, modality, adverse events, as well as the influence of genotype and dietary composition of CR [80].

Previous systematic reviews investigated the effect of CR on the development and progression of age-related chronic diseases in animal models [81,82,83] and humans, focusing on a limited number of health outcomes [84,85] or examining lifespan outcomes in cohorts not entirely representative of the general population [86]. Opposed to these previous studies, our systematic review was focused on the impact of CR on health predictors of longevity, disease risk factors and quality of life in the general population.

Our search identified 29 articles reporting data from eight RCTs with a total of 704 enrolled participants (67.9% women, 10.5% of lost at follow-up). Our findings suggest that CR can prompt a variety of favorable changes in adults across a considerable number of health predictors of quality of aging, such as anthropometric outcomes, body composition, energy homeostasis, oxidative stress and inflammation, cardiovascular disease, insulin sensitivity, mood disorders, well-being and quality of life.

All RCTs included in this analysis reported statistically significant improvements in anthropometric outcomes, namely BMI and fat mass, with an effect that was expectedly stronger in people diagnosed with overweight. CR also induced positive changes on total cholesterol, LDL, fasting insulin and glucose. Although the metabolic result could reflect improvements in body composition and chronic inflammation [87], we failed to document significant changes in inflammatory markers and insulin-sensitizing peptides, e.g., adiponectin and ghrelin, likely due to the small number of available studies for meta-analysis. With regards to energy-related hormones, CR favored an energy-sparing adaptive response involving leptin and, likely through its mechanistic link to the hypothalamic–pituitary–thyroid axis, of thyroid hormones, these latter controlling several aging accelerators, such as mitochondrial activity, heat production and inflammation [88]. In terms of cardiovascular risk, we collected discordant results on blood pressure and observed a reduction in SBP and DBP only in meta-analysis (sensitivity analysis, Table 2 in Table S3 Table of results), which could have been influenced by the short follow-up [15] or the small sample size [57].

Some studies were balanced at baseline not only for BMI, sex, fat mass, weight, but also for blood pressure, lipid profile, and glucose/insulin; on the other hand, other studies were not balanced for these last characteristics. In addition, heterogeneity of protocols of RCTs included (as the follow-up lengths or some characteristics of population observed like the obesity) could have influenced results summarized with meta-analysis. So, we performed meta-analyses by subgroups to control possible confounding factors in quantitative synthesis.

Together, our findings recollect and consolidate current evidence on the link between CR and markers relating to health-span and longevity. Hypothetically, these results could hint at the therapeutic implications of CR in non-pharmacological management of a many chronic diseases and cardiometabolic problems originating from inadequate lifestyle habits. However, additional evidence is needed to support to clinical practice and/or prevention and health promotion during lifespan. This SR underlined that, excluding body composition, blood lipids and blood pressure, trials reporting data on hormones and glucose metabolism, osteoporosis and bone health and inflammatory markers are few to support strong conclusion in favor to caloric restriction for healthy aging. At the same time, the possibility of a next increase of knowledge on this topic is probably very close thanks to the elevated number of recent trials in recruitment phase that we found from our search (*n* = 58).

In exploring indicators linked to multiple dimensions of health, we examined outcomes relating to well-being and quality of life and documented marginal effects of CR on vitality and mood disorders, perception of stress and sleep quality [53,75]. Because studies were focused on intermediate outcomes as endpoints of the CR dietary pattern, we were unable to distinctively identify CR effects on physical, behavioral, cognitive or social functionality or risk of onset of diseases. Likewise, data retrievable on CR effects on bone health were scant [69]. Collectively, current findings can only hint toward a soothing impact of CR on other dimensions of the health, overall aggregated under the umbrella of multidimensional aspects of health.

This SR has some potential limitations that should be mentioned. First, this SR and meta-analyses included a small number of trials about association between CR and some healthy aging outcomes. Hence, our results need to be upgraded with new evidence about the observed associations. Second, the studies included in the SR were mostly conducted in North American populations, such that results may not be entirely applicable to non-US populations, with different eating patterns, levels of physical activity and average BMI. Third, the heterogeneity of indicators and outcomes as well as the type of data reported in articles did not allow us to meta-analyze all available results. Fourthly, although the Cochrane Tool showed good methodological quality within the selected studies, some RCTs were at high risk of bias. One key limit of our SR is the overall small sample of participants in the studies herein included, which precluded further analysis stratifications and, likely, explained the lack of significance of some estimates of the meta-analysis (i.e., SBP, DBP and HDL). Lastly, the asymmetry in funnel plots and the presence of RCTs registered in ClinicalTrial.gov without publication cast doubts on potential publication bias, thereby suggesting that CR effectiveness found in the review could be overestimated. We attempted to mitigate this potential publication bias by including adherence to PRISMA statement for design, conduction and reporting of SR and meta-analyses, as well as double-blind selection and quality evaluation processes. The aim of our meta-analysis was to understand if CR improves health, increases lifespan and is safe in people without diseases apart overweight. We showed that CR of 10–25% of the total caloric intake was efficacious on some markers of health relatively to metabolism, endocrine system, and inflammation without main adverse events. However, these findings are limited to a short period of observation, in people mostly aged 70 years, and overweight. The effect on normal-weight subjects, the potential deprivation of nutritional agents on the long-term, and the need of food supplement are not investigated at all. Although increasing data on animals are encouraging, there is not enough evidence to suggest CR for a long time at any age, any weight, any diseases. On the other hand, a period of CR of 10–25% of the total caloric intake for 12–24 months could be suggested in mid-age overweight people to improve weight and related cardiometabolic dysfunctions as an alternative to classical weight-loss programs.

## 5. Conclusions

Taken together, CR of 10% or more of the total caloric intake can improve many risk factors in the adult population. CR impact on markers of cardiometabolic health appears to foster the usefulness of CR for chronic age-related diseases, such as cardiovascular diseases and diabetes. Calorie-restricted dietary pattern also showed to improve inflammatory status, hormone profiles and, albeit poorly investigated, outcomes relating to mood and quality of life. Nevertheless, slight declines in bone density and lean body mass occur after CR, and changes in arterial blood pressure and HDL-cholesterol levels seem less responsive to CR. Limited evidence also exists on the impact of CR in the multidimensional process of health inclusive of social participation and function, as well as physical or cognitive functionality.

Unfortunately, the relatively small sample of studies and the short duration of follow-ups precluded the outlining of a clear relationship between CR and the development of chronic diseases, nor did current finding allow to profile a “golden” calorie restriction to improve cardiometabolic health on the long term at any age and weight after the regime has ceased. Further well-designed controlled trials, with adequate sample sizes, normal-weight subjects and longer follow-ups are warranted to better investigate the mechanisms involved in CR effects on health outcomes and the safety of a prolonged regimen. There is not yet enough strong evidence to suggest to the public CR. 

## Figures and Tables

**Table 1:** Characteristics of included studies. Main baseline characteristics of randomized controlled trials (RCTs) included in systematic review (SR) (further details are reported in Table S3 Table of results).

First Author, Year of Publication	Country	Duration (Months)	Per protocol Caloric Restriction (% kcal)	Participants (int/ctrl)	Females (int/ctrl)	Mean Age (yr)	BMI (int/ctrl)	Extrapolated Outcomes	Inclusion of Healthy Participants
Armamento-Villareal 2012	USA	12	Females: 31–47% * Males: 25–38% *	26/27	18/17	75	37.2/37.3	BMI, BC, BM, QoL	no
Buchowski 2012	USA	1	25%	32/8	32/8	31.5	32.2/30.1	BMI, BC, ABP, HP, lipids, IM	yes
Haas 2014	USA	12	Females: 31% * Males: 25% *	55/54	32/37	70.3	30–40 **	BMI, BC, ABP, HP, lipids, IM	yes
Heilbronn 2006	USA	6	25%	12/11	6/5	38.5	27.8 ***	BMI, BC, ABP, HP, GM, lipids, BM, IM, OM	yes
Racette 2006	USA	12	16–20%	18/10	12/6	55.6	27.1/27.9	BMI, ABP, HP, GM, lipids, IM	yes
Ravussin 2015	USA	24	25%	143/75	99/53	35	25.2/25.1	BMI, BC, HP, GM, lipids, IM, QoL, MI, SQ, SxF	yes
Sparks 2016	USA	24	25%	33/18	23/11	39	25.3/25.1	BMI, ABP, GM, lipids, OM	yes
Teng 2011	Malaysia	3	15–25%	14/14	0/0	58.8	27.0/26.5	BMI, BC, QoL, MI, SQ	yes

For abbreviations: *—CR estimated assuming a daily energy requirement of 1600 kcal (females) and 2000 kcal—(males); **—BMI range; ***—average BMI value; int/ctrl—intervention/control; ABP—arterial blood pressure; BC—body composition; BMI—body mass index; GM—glucose metabolism; HP—hormone profile; IM—inflammatory markers; MI—mood indices; OM—oxidative markers; QoL—quality of life; SQ—sleep quality; SxF—sexual function.

**Table 2:** Risk of Bias within included RCTs. We used the Cochrane’s risk of bias tool [61]. Each dimension was evaluated by two blinding reviewers, with results of concordance being here presented.

Author	Selection Bias (Randomization)	Selection Bias (Allocation Concealment)	Performance Bias Objective Outcomes	Performance Bias Subjective Outcomes	Detection Bias Objective Outcomes	Detection Bias Subjective Outcomes	Attrition Bias	Reporting Bias	Other Bias	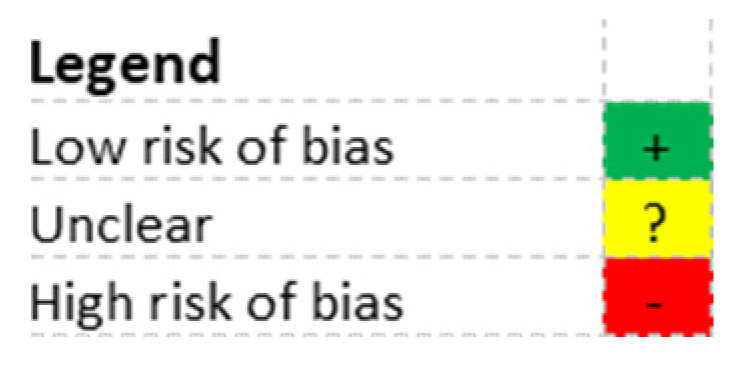
Armamento-Villareal 2012	+	+	+		+		+	+	+	
Buchowski 2012	+	−	+		+		+	+	+	
Haas 2014	+	+	+		+		?	+	−	
Heilbronn 2006	+	+	+		+		−	−	−	
Racette 2006	+	+	+		+		−	−	−	
Ravussin 2015	+	−	+	−	+	−	+	+	+	
Sparks 2016	+	+	+		+		+	+	?	
Teng 2011	?	?	+	−	+	−	+	−	−	

**Table 3:** Meta-analysis comparing CR regimens with control: mean difference (MD) 95% CI and I-squared percentages for weight, fat mass and body mass index (BMI).

Outcome	By Subgroup Meta-Analysis		No. of Studies	MD (95% CI)	I^2^ (%)
Weight *	Overall		6	−7.90 (−7.99, −7.81)	0.0
		Normal weight	2	−7.90 (−7.99, −7.81)	0.0
	By BMI	Overweight	3	−6.50 (−10.61, −2.40)	0.0
		Obese	1	−3.30 (−17.72, 11.12)	–
	By follow-up	≤5 months from baseline	2	−4.26 (−9.33, −0.80)	0.0
		≥6–≤11 months from the baseline	1	−8.70 (−17.36, −0.04)	–
		≥12 months from the baseline	3	−7.90 (−7.99, −7.81)	0.0
Fat mass *	Overall		5	−4.40 (−6.69, −0.45)	85.7
	By follow-up	≤6 months from baseline	2	−1.91 (−3.37, −0.45)	0.0
		>6 months from baseline	3	−5.80 (−5.87, −5.72)	0,0
	By BMI	Normal weight	2	−5.80 (−5.87, −5.72)	0.0
		Overweight	2	−3.64 (−7.70, −0.41)	76.5
		Obese	1	−2.40 (−12.72, 7.92)	–
BMI **	Overall		5	−2.68 (−3.51, −1.86)	69.6
	By follow-up	Follow-up ≤5 months from baseline	1	−0.50 (−1.91, 0.91)	–
		Follow-up ≥6–≤11 months from the baseline	1	−4.30 (−6.18, −2.42)	–
		Follow-up ≥12 months from the baseline	3	−2.70 (−2.73, −2.67)	0.0

* MD is expressed in kg; ** MDs are expressed in kg/m^2^.

## Supplementary Materials

The following are available online at https://www.mdpi.com/2072-6643/12/8/2290/s1, Figure S1: Funnel plots for publication bias, Table S1: Search strategy, Table S2: List of studies included, Table S3: Table of results.
