# Supplementary material for: Is Caloric Restriction Associated with Better Healthy Aging Outcomes? A Systematic Review and Meta-Analysis of Randomized Controlled Trials
Source: Nutrients. 2020 Jul 30;12(8):2290. doi: 10.3390/nu12082290 (PMC7468870; doi:10.3390/nu12082290)
Supplement: Supplementary file 1 [file nutrients-12-02290-s001.zip › Figure S1 Forest and funnel plots.docx]

**Figure S1 Forest and Funnel plots**


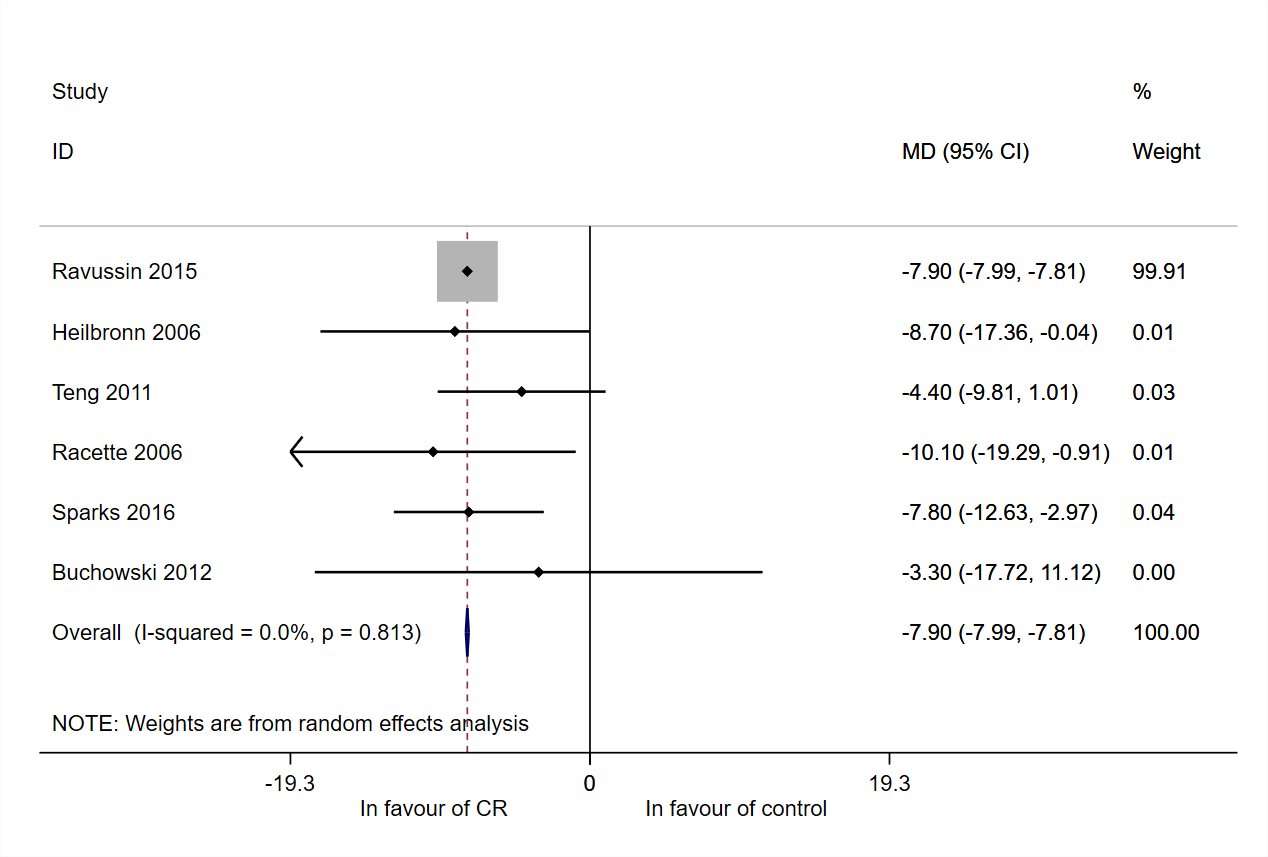


**Figure 1. Meta-analysis with random effects method, Outcome Weight.** Meta-analysis was performed using post mean value (or median value) or mean change within groups and calculating Mean Differences (with their 95% CI). All estimates are expressed in kg.


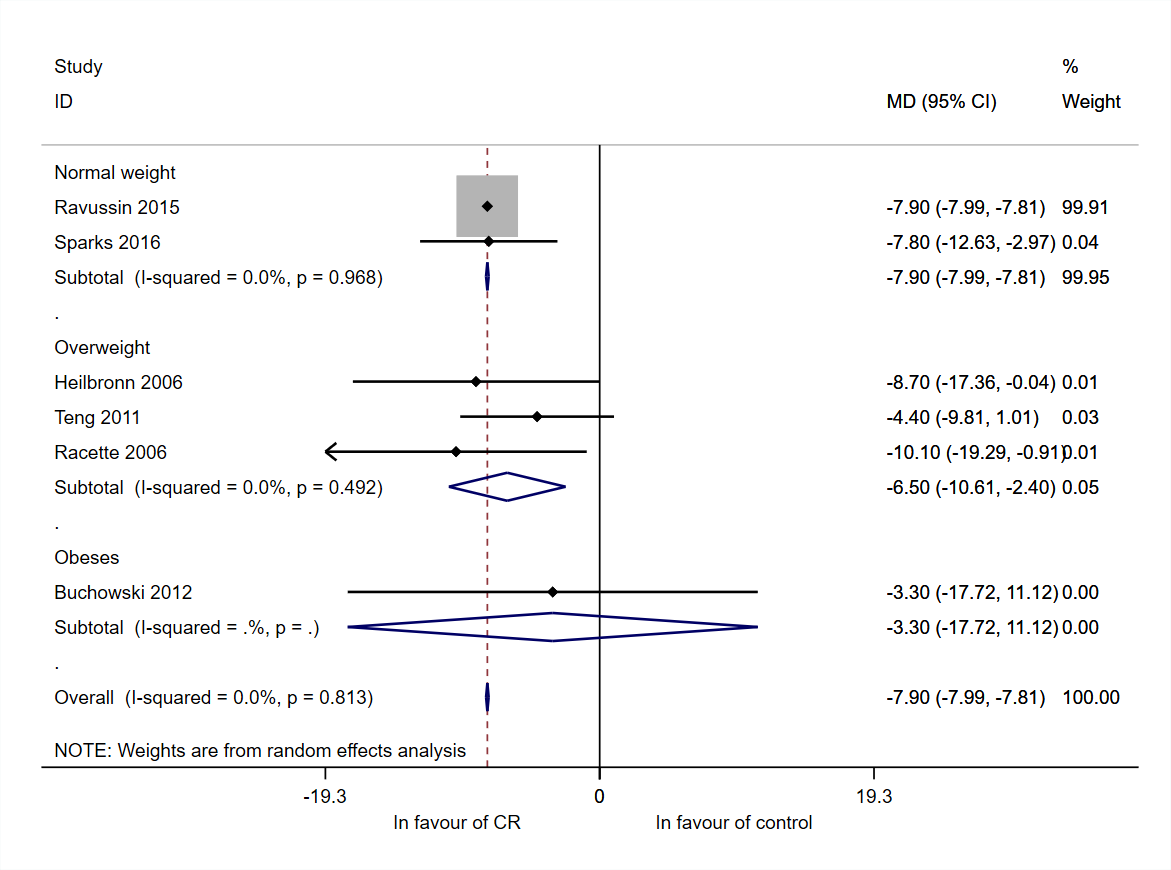


**Figure 2. Meta-analysis with random effects method, Outcome Weight by BMI.** Meta-analysis was performed using post mean value (or median value) or mean change within groups and calculating Mean Differences (with their 95% CI). All estimates are expressed in kg.


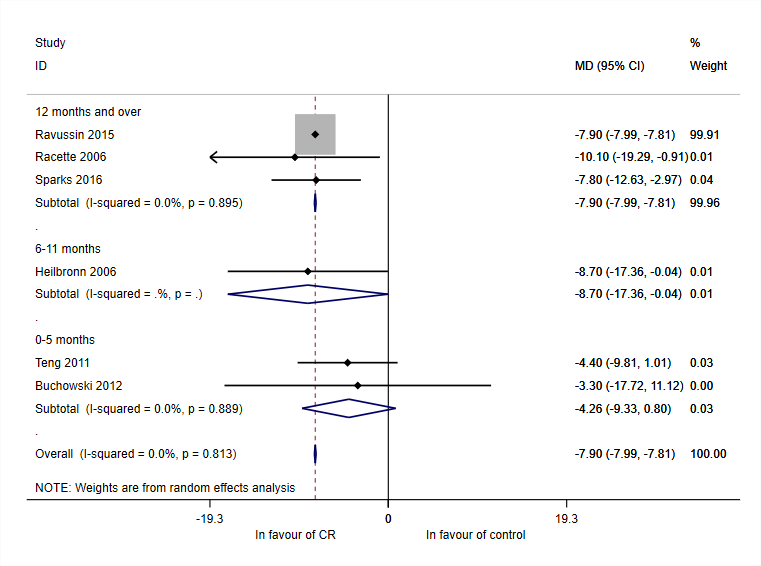


**Figure 3. Meta-analysis with random effects method, Outcome Weight by follow-up.** Meta-analysis was performed using post mean value (or median value) or mean change within groups and calculating Mean Differences (with their 95% CI). All estimates are expressed in kg.


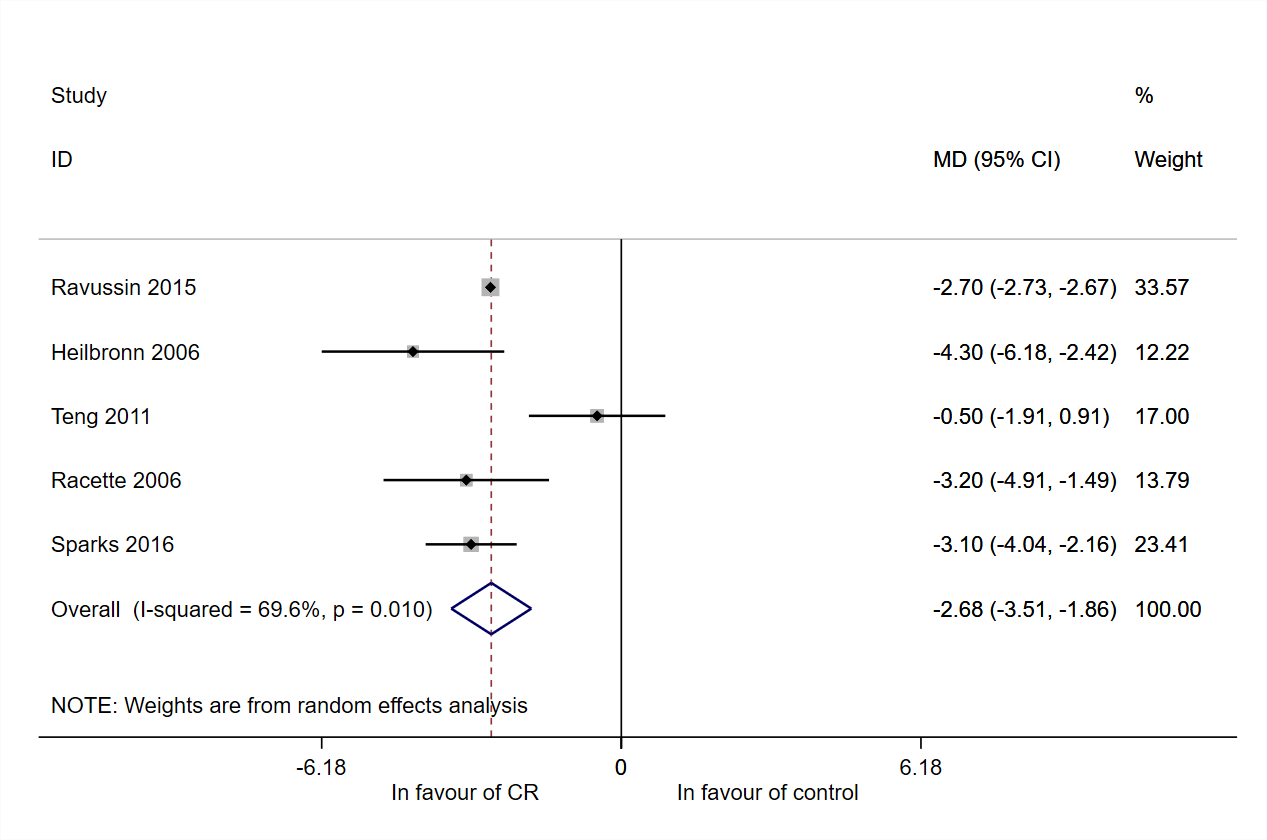


**Figure 4. Meta-analysis with random effects method, Outcome BMI.** Meta-analysis was performed using post mean value (or median value) or mean change within groups and calculating Mean Differences (with their 95% CI). All estimates are expressed in kg/m^2^.


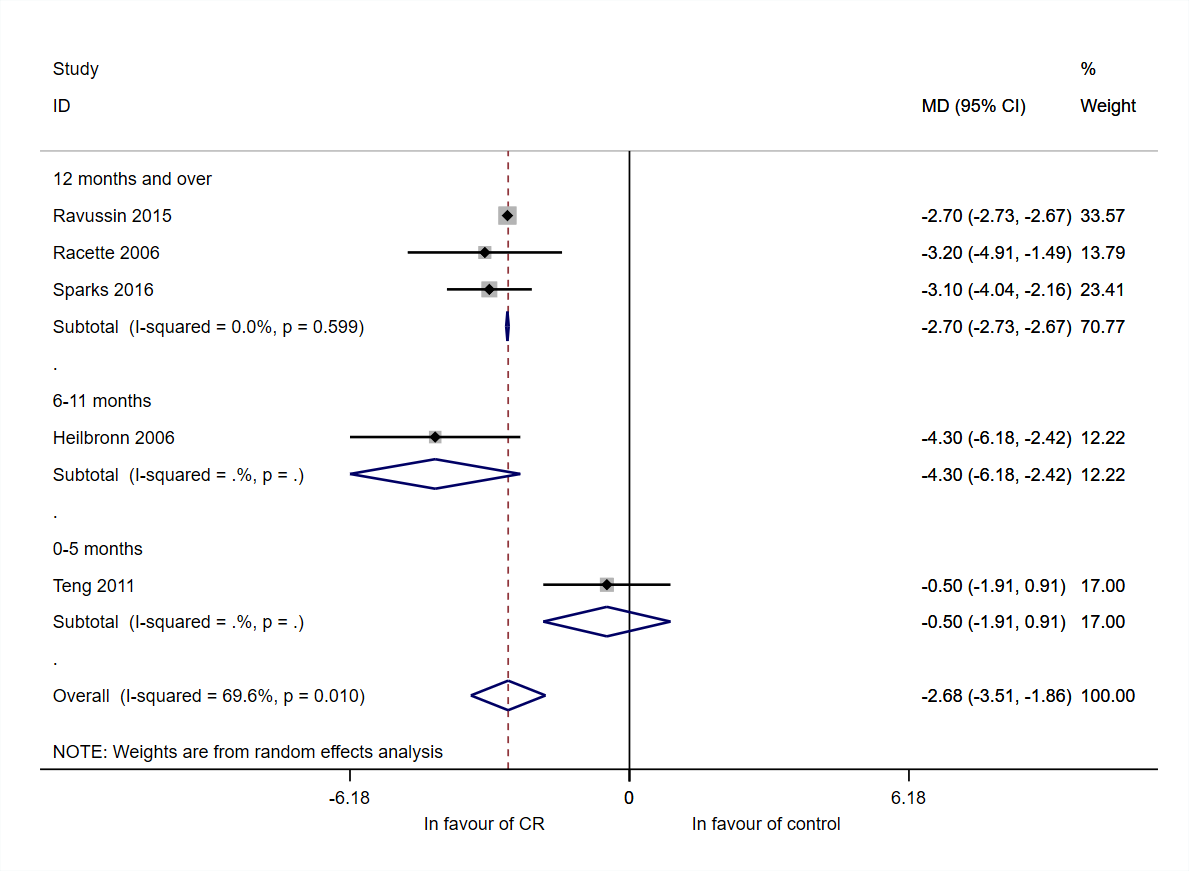


**Figure 5. Meta-analysis with random effects method, Outcome BMI by follow-up.** Meta-analysis was performed using post mean value (or median value) or mean change within groups and calculating Mean Differences (with their 95% CI). All estimates are expressed in kg/m^2^.


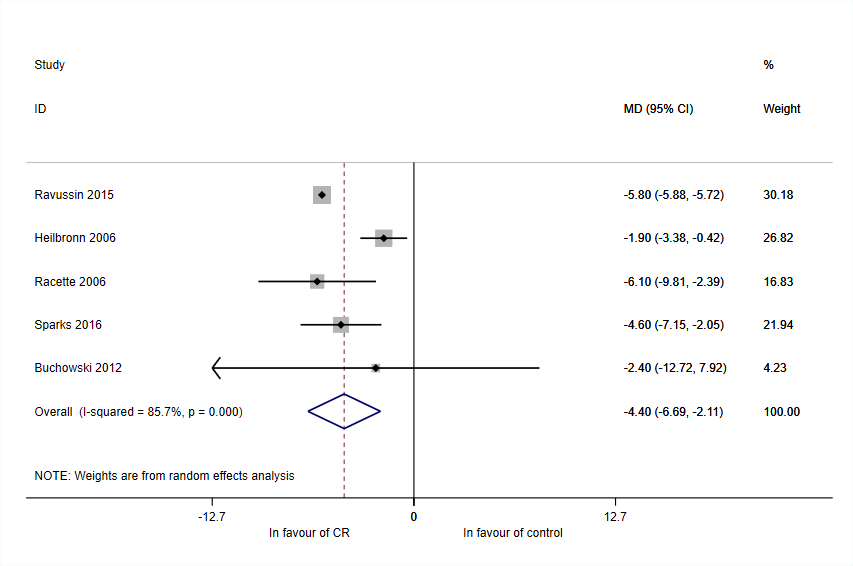


**Figure 6. Meta-analysis with random effects method, Outcome Fat mass.** Meta-analysis was performed using post mean value (or median value) or mean change within groups and calculating Mean Differences (with their 95% CI). All estimates are expressed in kg.


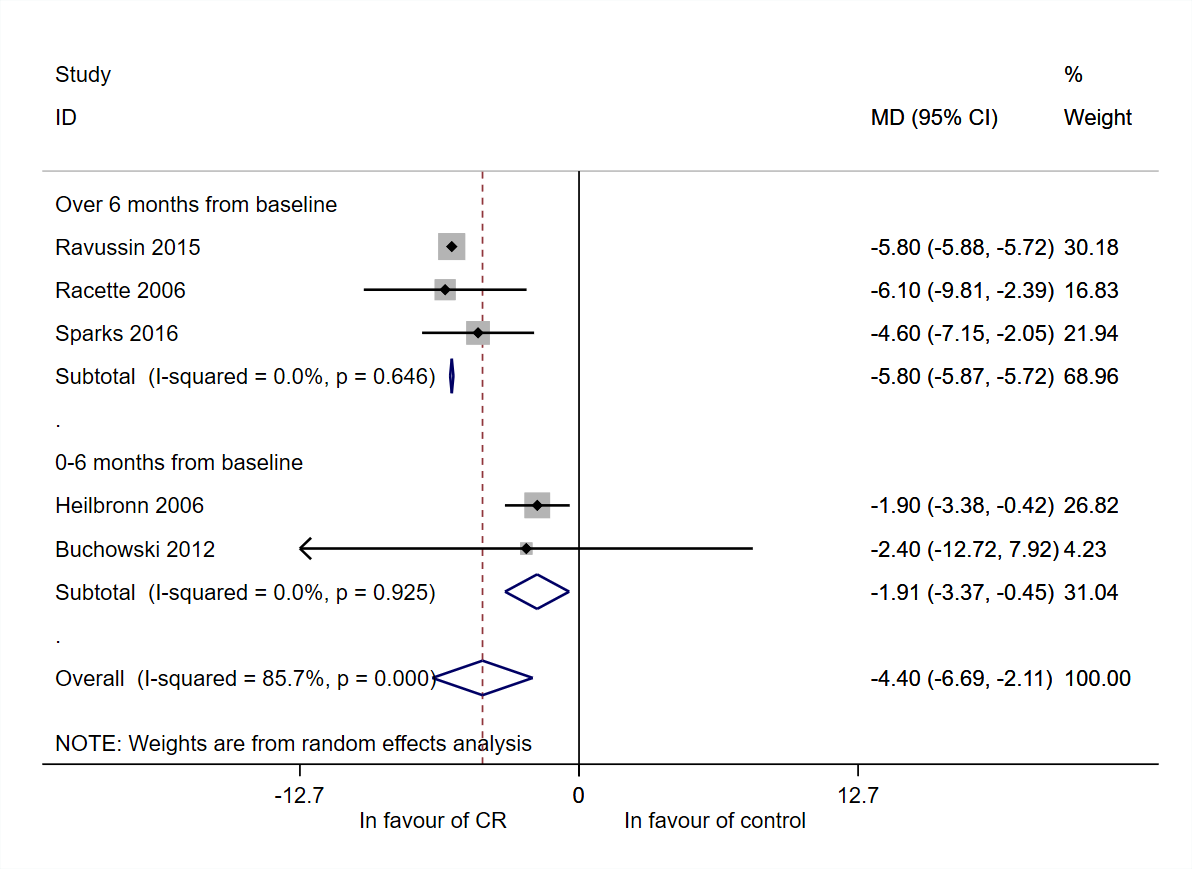


**Figure 7. Meta-analysis with random effects method, Outcome Fat mass by follow-up.** Meta-analysis was performed using post mean value (or median value) or mean change within groups and calculating Mean Differences (with their 95% CI). All estimates are expressed in kg.


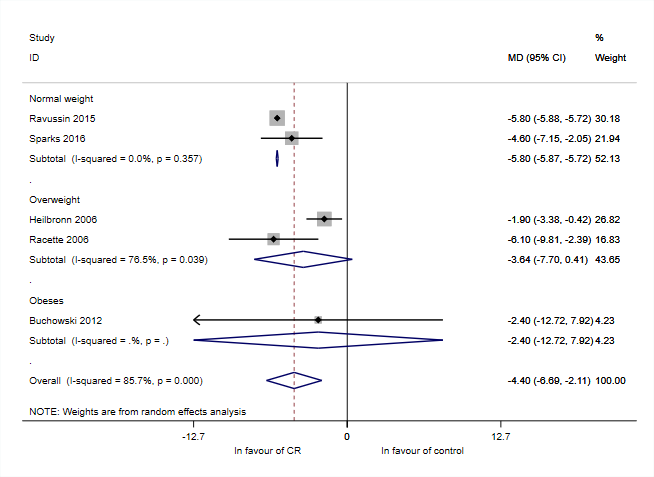


**Figure 8. Meta-analysis with random effects method, Outcome Fat mass by BMI.** Meta-analysis was performed using post mean value (or median value) or mean change within groups and calculating Mean Differences (with their 95% CI). All estimates are expressed in kg.


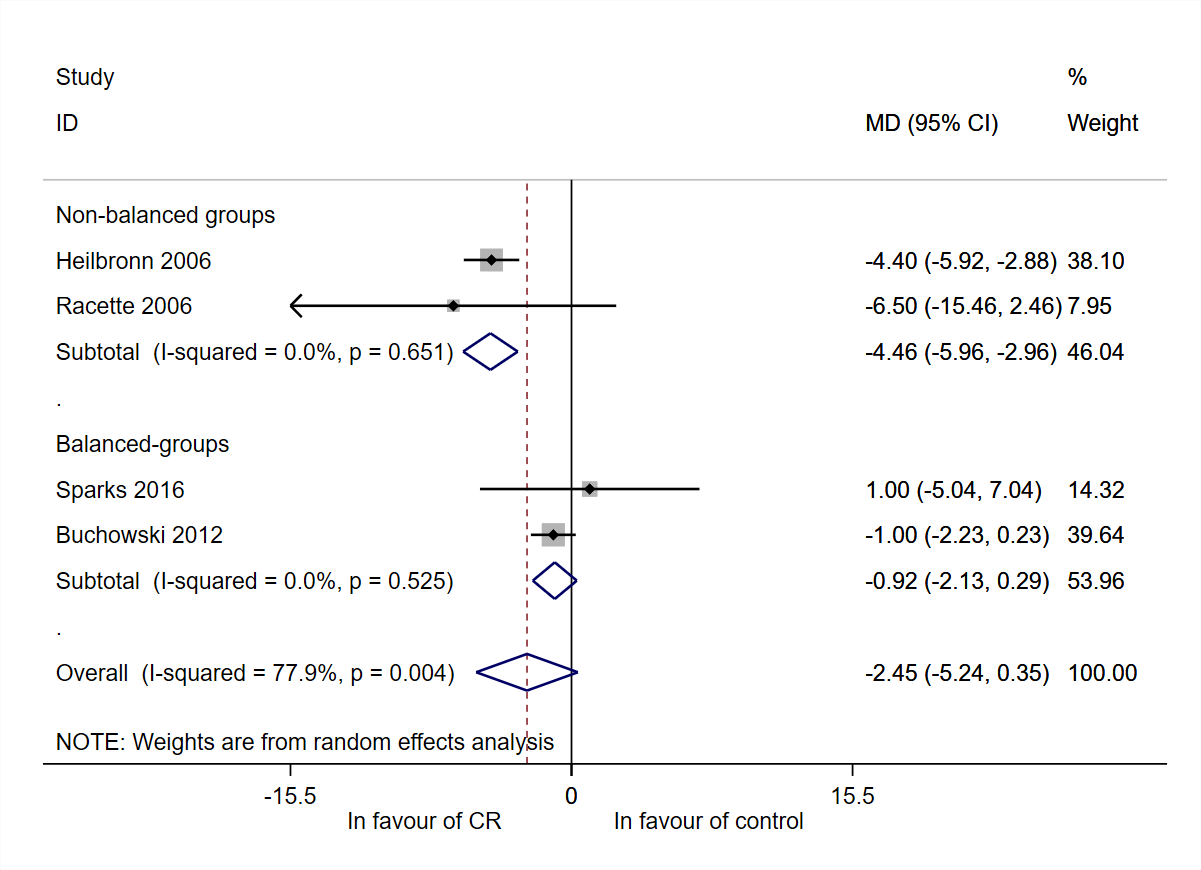


**Figure 9. Sensitivity analysis: meta-analysis with random effects method, Outcome SBP by balance of groups at baseline.** Meta-analysis was performed using post mean value (or median value) or mean change within groups and calculating Mean Differences (with their 95% CI). All estimates are expressed in mmHg.


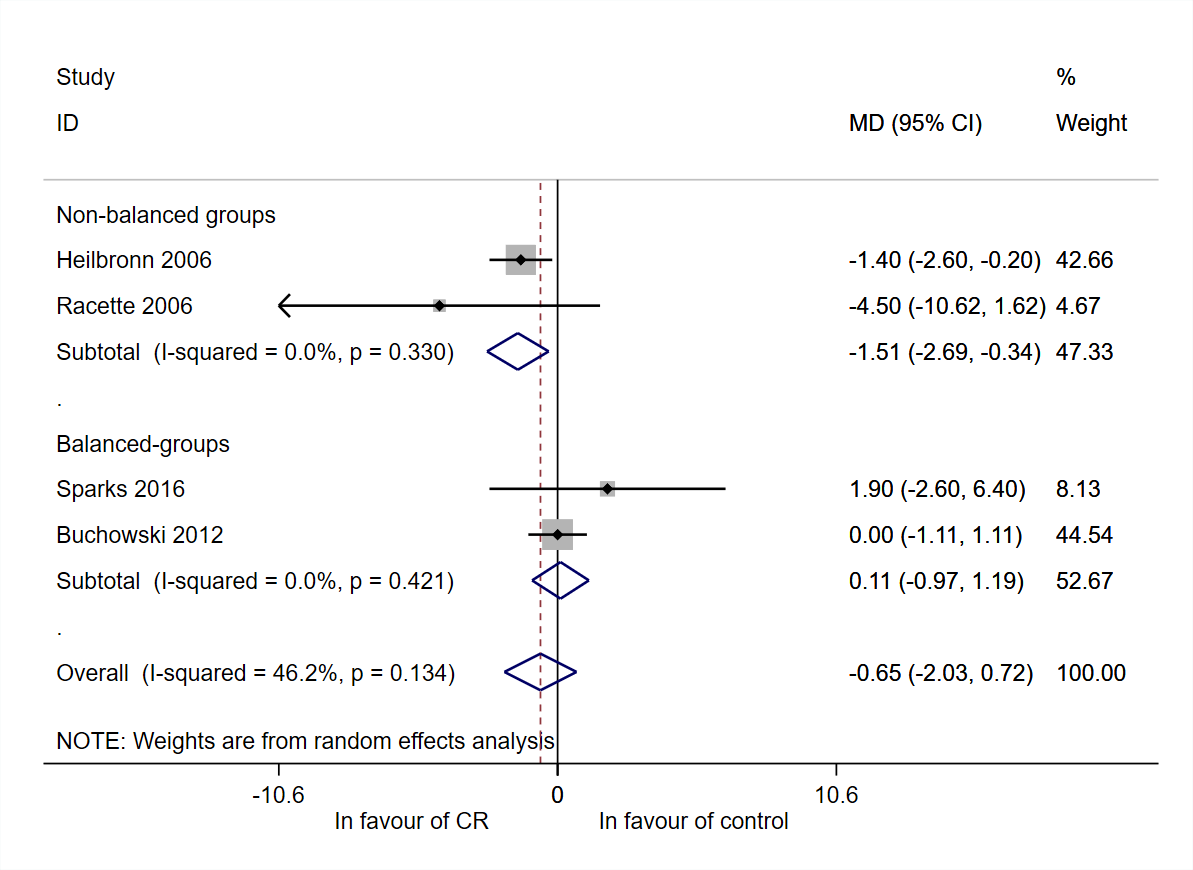


**Figure 10. Sensitivity analysis: meta-analysis with random effects method, Outcome DBP by balance of groups at baseline.** Meta-analysis was performed using post mean value (or median value) or mean change within groups and calculating Mean Differences (with their 95% CI). All estimates are expressed in mmHg.


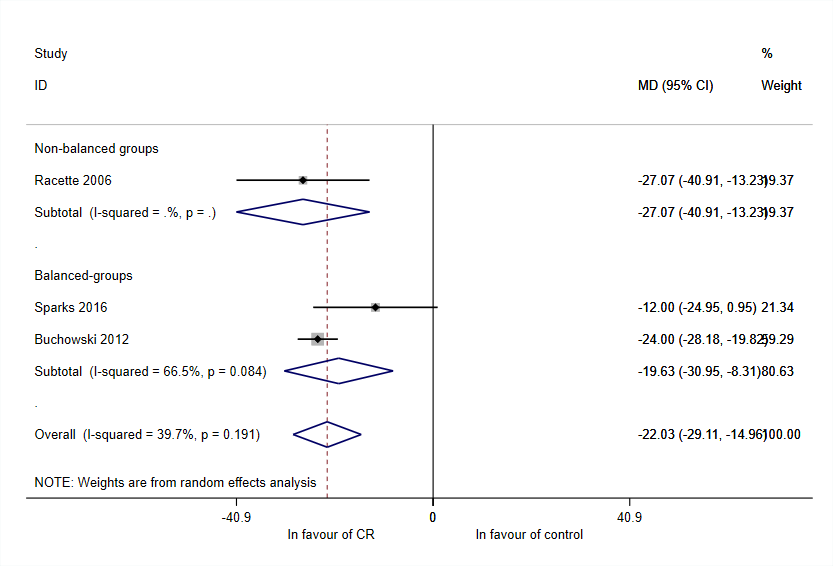


**Figure 11. Sensitivity analysis: meta-analysis with random effects method, Outcome LDL by balance of groups at baseline.** Meta-analysis was performed using post mean value (or median value) or mean change within groups and calculating Mean Differences (with their 95% CI). All estimates are expressed in mg/dl.


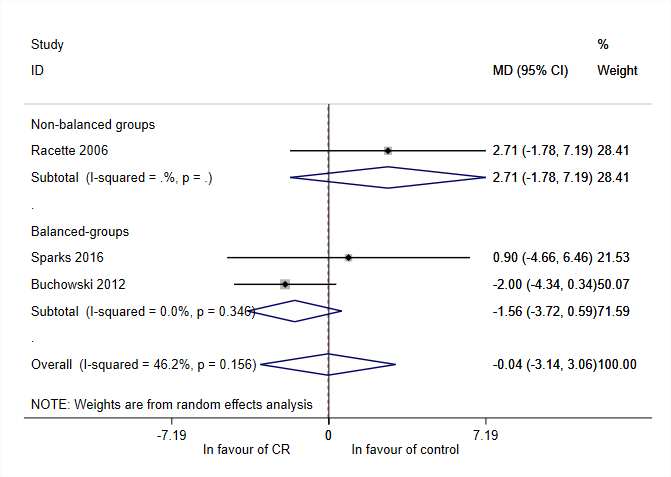


**Figure 12. Sensitivity analysis: meta-analysis with random effects method, Outcome HDL by balance of groups at baseline.** Meta-analysis was performed using post mean value (or median value) or mean change within groups and calculating Mean Differences (with their 95% CI). All estimates are expressed in mg/dl.


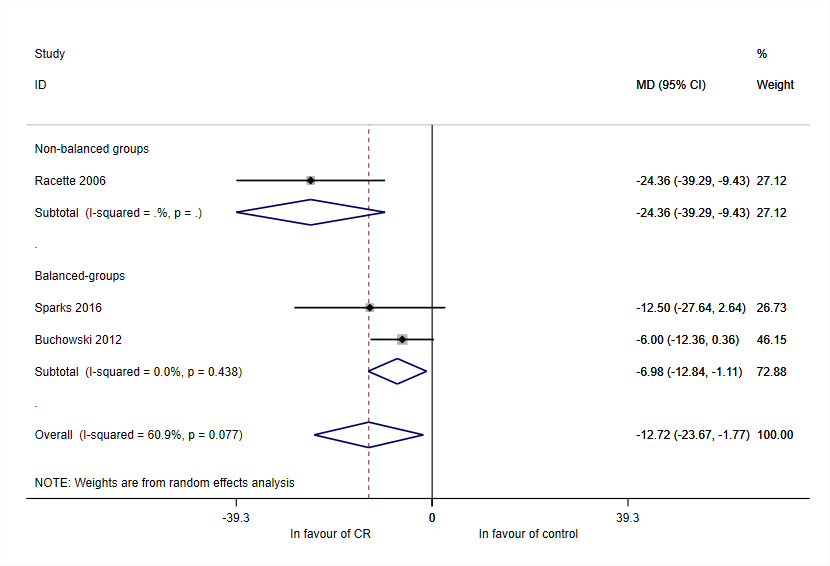


**Figure 13. Sensitivity analysis: meta-analysis with random effects method, Outcome Total cholesterol by balance of groups at baseline.** Meta-analysis was performed using post mean value (or median value) or mean change within groups and calculating Mean Differences (with their 95% CI). All estimates are expressed in mg/dl.


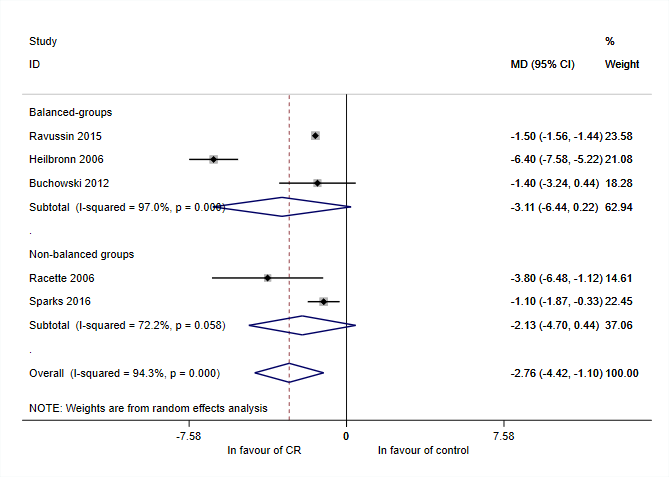


**Figure 14. Sensitivity analysis: meta-analysis with random effects method, Outcome Fasting insulin by balance of groups at baseline.** Meta-analysis was performed using Mean Differences (with their 95% CI). All estimates are expressed in mIU/L.


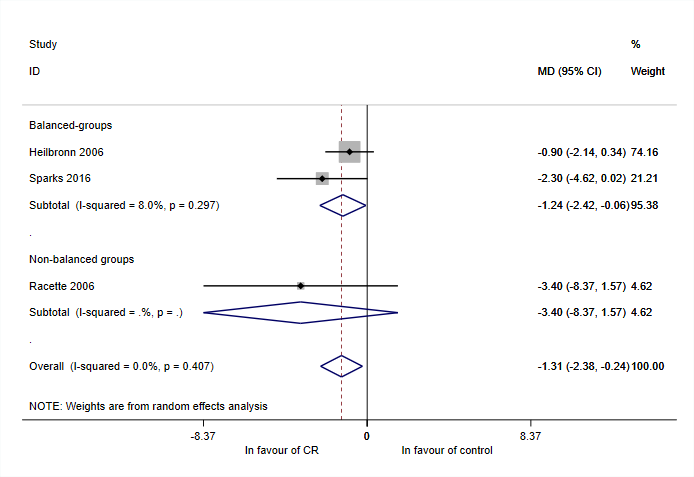


**Figure 15. Sensitivity analysis: meta-analysis with random effects method, Outcome Fasting glucose by balance of groups at baseline.** Meta-analysis was performed using Mean Differences (with their 95% CI). All estimates are expressed in mg/dl.


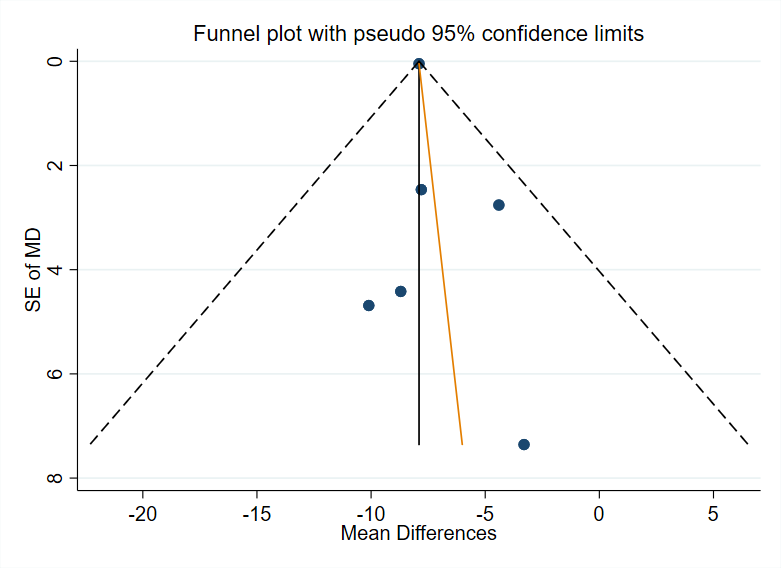


**Figure 16.** **Funnel plot 1, Outcome Weight.** Verification of publication bias in the meta-analysis of effect sizes of outcome *Weight*.


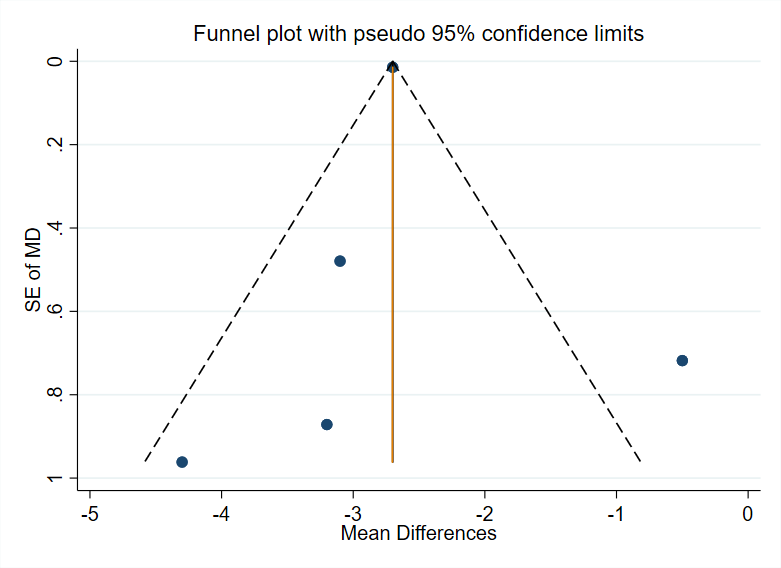


**Figure 17**. **Funnel plot 2, Outcome BMI.** Verification of publication bias in the meta-analysis of effect sizes of outcome *BMI*.


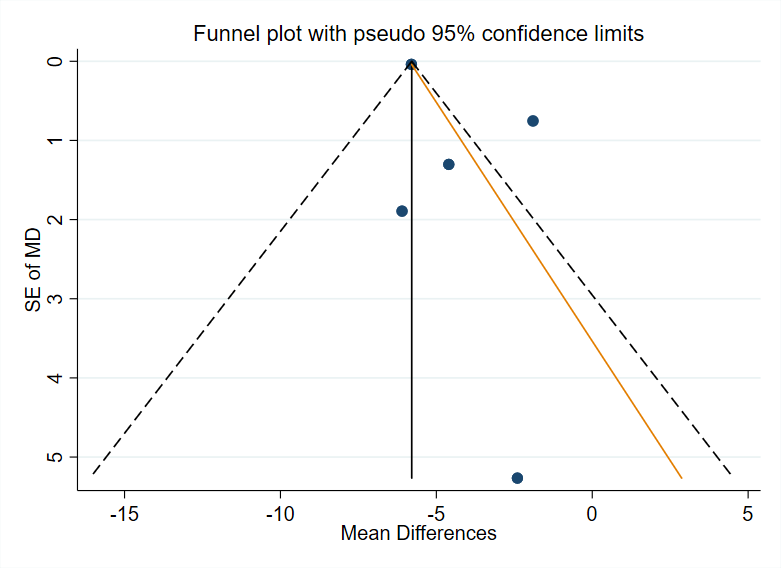


**Figure 18.** **Funnel plot 3, Outcome Fat mass.** Verification of publication bias in the meta-analysis of effect sizes of outcome *Fat mass.*


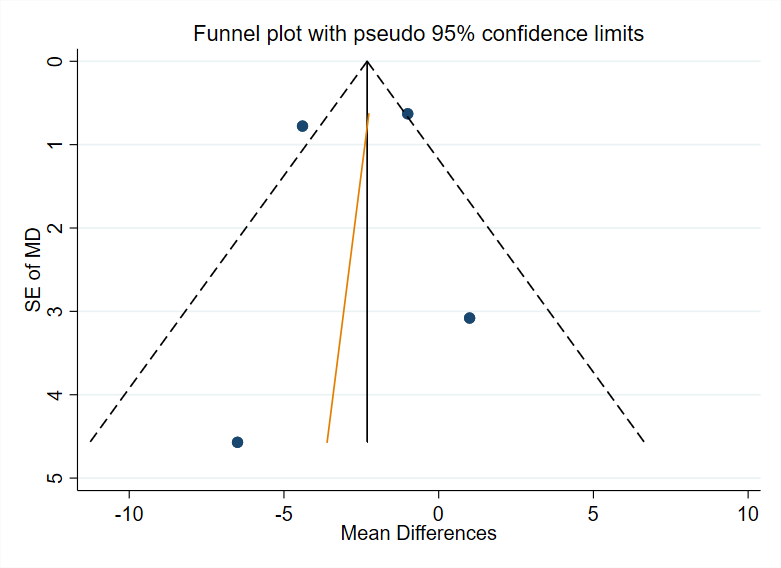


**Figure 19. Funnel plot 4, Outcome Systolic Blood Pressure.** Verification of publication bias in the meta-analysis of effect sizes of outcome *Systolic blood pressure*.


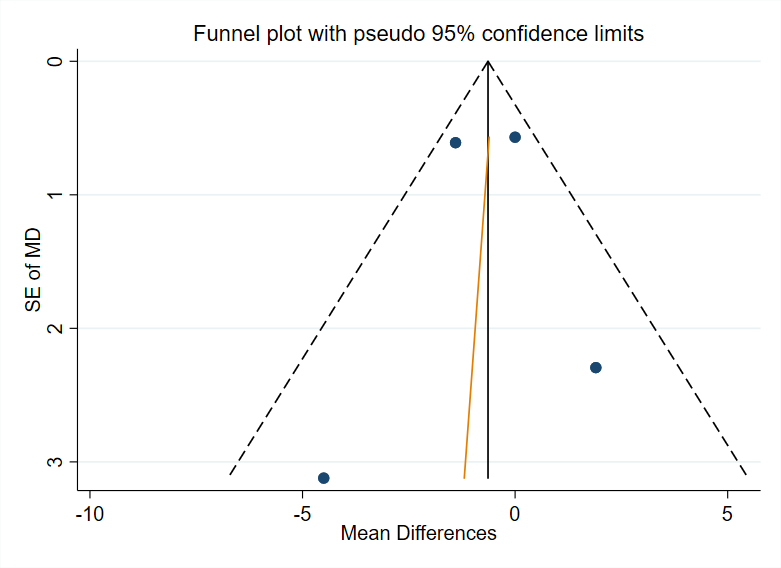


**Figure 20**. **Funnel plot 5, Outcome Diastolic Blood Pressure.** Verification of publication bias in the meta-analysis of effect sizes of outcome *Diastolic blood pressure*.


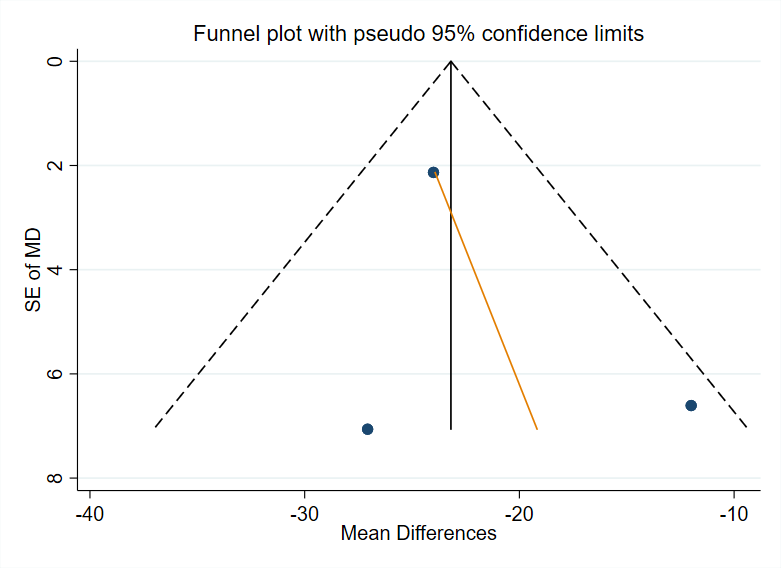


**Figure 21**. **Funnel plot 6, Outcome LDL.** Verification of publication bias in the meta-analysis of effect sizes of outcome *LDL*.


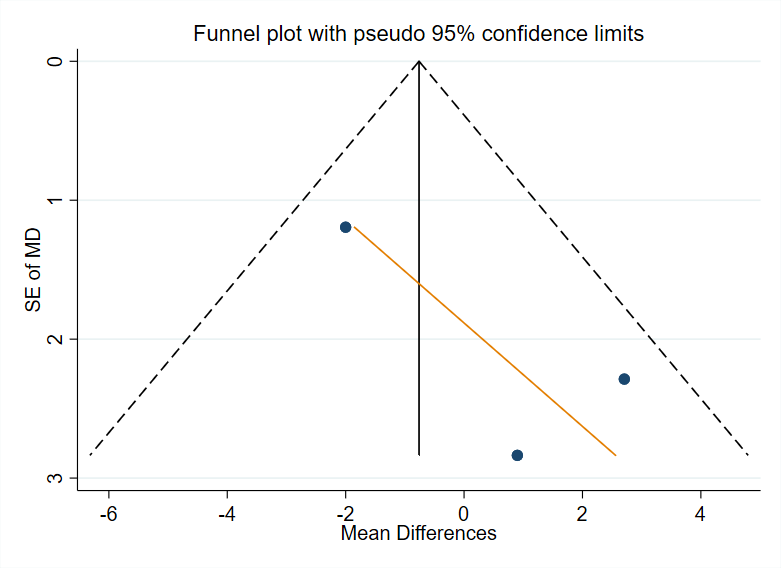


**Figure 22. Funnel plot 7, Outcome HDL.** Verification of publication bias in the meta-analysis of effect sizes of outcome *HDL.*


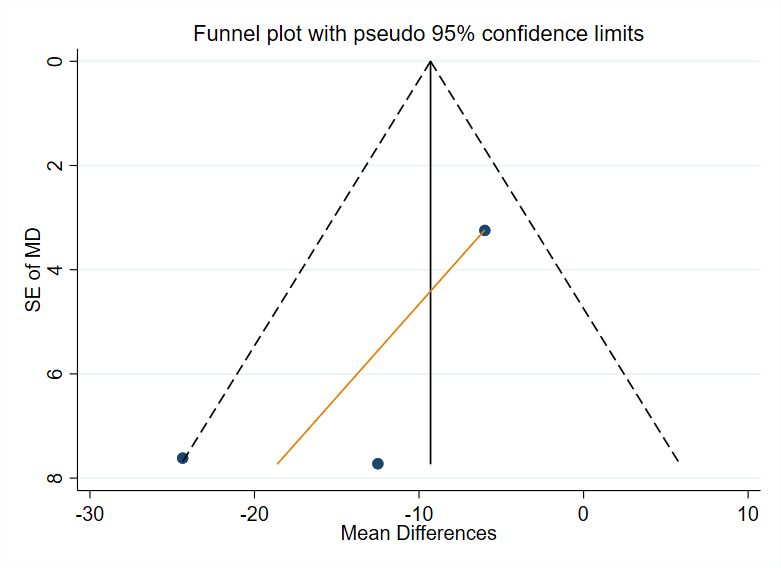


**Figure 23**. **Funnel plot 8, Outcome Total Cholesterol.** Verification of publication bias in the meta-analysis of effect sizes of outcome *Total cholesterol*.


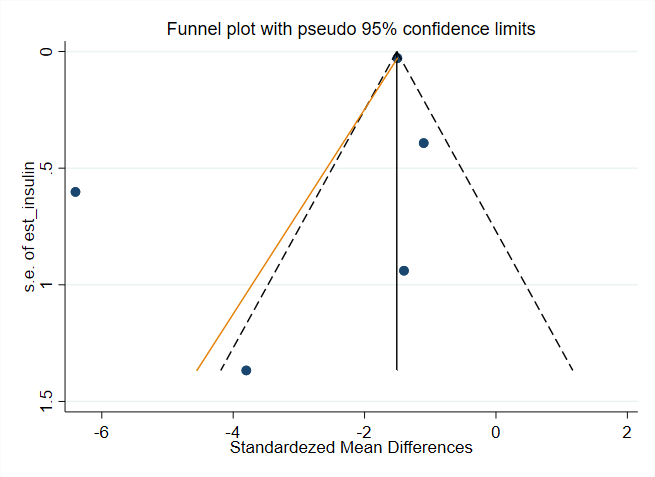


**Figure 24. Funnel plot 9, Outcome Fasting insulin.** Verification of publication bias in the meta-analysis of effect sizes of outcome *Fasting* *insulin*.


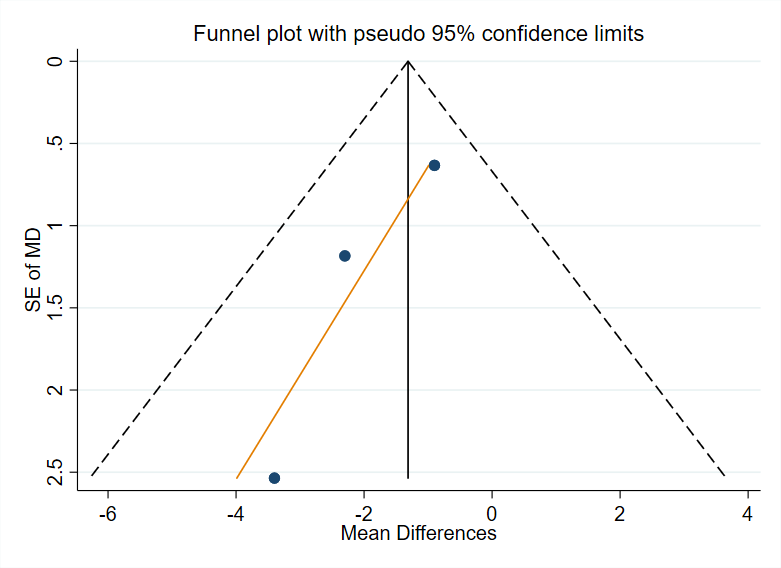


**Figure 25**. **Funnel plot 10, Outcome Fasting glucose.** Verification of publication bias in the meta-analysis of effect sizes of outcome *Fasting glucose*.
