# Supplementary material for: Is Caloric Restriction Associated with Better Healthy Aging Outcomes? A Systematic Review and Meta-Analysis of Randomized Controlled Trials
Source: Nutrients. 2020 Jul 30;12(8):2290. doi: 10.3390/nu12082290 (PMC7468870; doi:10.3390/nu12082290)
Supplement: Supplementary file 1 [file nutrients-12-02290-s001.zip › Table S1 Search strategy.docx]

**Table S1 Search strategy**

**1) Search 1**

**Database** PubMed

**Date** 31^st^ March, 2019

**Total records** 182

**Strings:**

#1, Search (((caloric restriction"[MeSH Terms] AND "aging"[MeSH Terms] AND "humans"[MeSH Terms]) AND "humans"[MeSH Terms])

#2, Search((("diet, reducing"[MeSH Terms] OR ("diet"[All Fields] AND "reducing"[All Fields]) OR "reducing diet"[All Fields] OR ("low"[All Fields] AND "calorie"[All Fields] AND "diet"[All Fields]) OR "low calorie diet"[All Fields] OR "caloric restriction"[MeSH Terms] OR ("caloric"[All Fields] AND "restriction"[All Fields]) OR "caloric restriction"[All Fields] OR ("low"[All Fields] AND "calorie"[All Fields] AND "diet"[All Fields])) OR (("lipids"[MeSH Terms] OR "lipids"[All Fields] OR "lipid"[All Fields]) AND restriction[All Fields]) OR fat-reduction[All Fields]) AND (("aging"[MeSH Terms] OR "aging"[All Fields]) OR anti-aging[All Fields] OR ("healthy aging"[MeSH Terms] OR ("healthy"[All Fields] AND "aging"[All Fields]) OR "healthy aging"[All Fields] OR ("healthy"[All Fields] AND "ageing"[All Fields]) OR "healthy ageing"[All Fields]))) AND (("mortality"[Subheading] OR "mortality"[All Fields] OR "mortality"[MeSH Terms]) OR ("mortality"[Subheading] OR "mortality"[All Fields] OR "survival"[All Fields] OR "survival"[MeSH Terms]) OR ("metabolic diseases"[MeSH Terms] OR ("metabolic"[All Fields] AND "diseases"[All Fields]) OR "metabolic diseases"[All Fields]) OR ("diabetes mellitus"[MeSH Terms] OR ("diabetes"[All Fields] AND "mellitus"[All Fields]) OR "diabetes mellitus"[All Fields] OR "diabetes"[All Fields] OR "diabetes insipidus"[MeSH Terms] OR ("diabetes"[All Fields] AND "insipidus"[All Fields]) OR "diabetes insipidus"[All Fields]) OR ("alzheimer disease"[MeSH Terms] OR ("alzheimer"[All Fields] AND "disease"[All Fields]) OR "alzheimer disease"[All Fields] OR "alzheimer"[All Fields])) AND "humans"[MeSH Terms]

# 3, Search ((("caloric restriction"[MeSH Terms] AND "aging"[MeSH Terms] AND "humans"[MeSH Terms]) AND ((((("diet, reducing"[MeSH Terms] OR ("diet"[All Fields] AND "reducing"[All Fields]) OR "reducing diet"[All Fields] OR ("low"[All Fields] AND "calorie"[All Fields] AND "diet"[All Fields]) OR "low calorie diet"[All Fields] OR "caloric restriction"[MeSH Terms] OR ("caloric"[All Fields] AND "restriction"[All Fields]) OR "caloric restriction"[All Fields] OR ("low"[All Fields] AND "calorie"[All Fields] AND "diet"[All Fields])) OR (("lipids"[MeSH Terms] OR "lipids"[All Fields] OR "lipid"[All Fields]) AND restriction[All Fields]) OR fat-reduction[All Fields]) AND (("aging"[MeSH Terms] OR "aging"[All Fields]) OR anti-aging[All Fields] OR ("healthy aging"[MeSH Terms] OR ("healthy"[All Fields] AND "aging"[All Fields]) OR "healthy aging"[All Fields] OR ("healthy"[All Fields] AND "ageing"[All Fields]) OR "healthy ageing"[All Fields]))) AND (("mortality"[Subheading] OR "mortality"[All Fields] OR "mortality"[MeSH Terms]) OR ("mortality"[Subheading] OR "mortality"[All Fields] OR "survival"[All Fields] OR "survival"[MeSH Terms]) OR ("metabolic diseases"[MeSH Terms] OR ("metabolic"[All Fields] AND "diseases"[All Fields]) OR "metabolic diseases"[All Fields]) OR ("diabetes mellitus"[MeSH Terms] OR ("diabetes"[All Fields] AND "mellitus"[All Fields]) OR "diabetes mellitus"[All Fields] OR "diabetes"[All Fields] OR "diabetes insipidus"[MeSH Terms] OR ("diabetes"[All Fields] AND "insipidus"[All Fields]) OR "diabetes insipidus"[All Fields]) OR ("alzheimer disease"[MeSH Terms] OR ("alzheimer"[All Fields] AND "disease"[All Fields]) OR "alzheimer disease"[All Fields] OR "alzheimer"[All Fields]))) AND "humans"[MeSH Terms]) AND "humans"[MeSH Terms]

#4, Search(((("caloric restriction"[MeSH Terms] AND "aging"[MeSH Terms] AND "humans"[MeSH Terms]) AND ((((("diet, reducing"[MeSH Terms] OR ("diet"[All Fields] AND "reducing"[All Fields]) OR "reducing diet"[All Fields] OR ("low"[All Fields] AND "calorie"[All Fields] AND "diet"[All Fields]) OR "low calorie diet"[All Fields] OR "caloric restriction"[MeSH Terms] OR ("caloric"[All Fields] AND "restriction"[All Fields]) OR "caloric restriction"[All Fields] OR ("low"[All Fields] AND "calorie"[All Fields] AND "diet"[All Fields])) OR (("lipids"[MeSH Terms] OR "lipids"[All Fields] OR "lipid"[All Fields]) AND restriction[All Fields]) OR fat-reduction[All Fields]) AND (("aging"[MeSH Terms] OR "aging"[All Fields]) OR anti-aging[All Fields] OR ("healthy aging"[MeSH Terms] OR ("healthy"[All Fields] AND "aging"[All Fields]) OR "healthy aging"[All Fields] OR ("healthy"[All Fields] AND "ageing"[All Fields]) OR "healthy ageing"[All Fields]))) AND (("mortality"[Subheading] OR "mortality"[All Fields] OR "mortality"[MeSH Terms]) OR ("mortality"[Subheading] OR "mortality"[All Fields] OR "survival"[All Fields] OR "survival"[MeSH Terms]) OR ("metabolic diseases"[MeSH Terms] OR ("metabolic"[All Fields] AND "diseases"[All Fields]) OR "metabolic diseases"[All Fields]) OR ("diabetes mellitus"[MeSH Terms] OR ("diabetes"[All Fields] AND "mellitus"[All Fields]) OR "diabetes mellitus"[All Fields] OR "diabetes"[All Fields] OR "diabetes insipidus"[MeSH Terms] OR ("diabetes"[All Fields] AND "insipidus"[All Fields]) OR "diabetes insipidus"[All Fields]) OR ("alzheimer disease"[MeSH Terms] OR ("alzheimer"[All Fields] AND "disease"[All Fields]) OR "alzheimer disease"[All Fields] OR "alzheimer"[All Fields]))) AND "humans"[MeSH Terms]) AND (Clinical Trial[ptyp] AND ("2006/01/01"[PDAT] : "2019/03/31"[PDAT]) AND "humans"[MeSH Terms]) Filters: Clinical Trial Sort by PubblicationDate from 2006/01/01 to 2019/03/31

#5, Search((("caloric restriction"[MeSH Terms] AND "aging"[MeSH Terms] AND "humans"[MeSH Terms]) AND ((((("diet, reducing"[MeSH Terms] OR ("diet"[All Fields] AND "reducing"[All Fields]) OR "reducing diet"[All Fields] OR ("low"[All Fields] AND "calorie"[All Fields] AND "diet"[All Fields]) OR "low calorie diet"[All Fields] OR "caloric restriction"[MeSH Terms] OR ("caloric"[All Fields] AND "restriction"[All Fields]) OR "caloric restriction"[All Fields] OR ("low"[All Fields] AND "calorie"[All Fields] AND "diet"[All Fields])) OR (("lipids"[MeSH Terms] OR "lipids"[All Fields] OR "lipid"[All Fields]) AND restriction[All Fields]) OR fat-reduction[All Fields]) AND (("aging"[MeSH Terms] OR "aging"[All Fields]) OR anti-aging[All Fields] OR ("healthy aging"[MeSH Terms] OR ("healthy"[All Fields] AND "aging"[All Fields]) OR "healthy aging"[All Fields] OR ("healthy"[All Fields] AND "ageing"[All Fields]) OR "healthy ageing"[All Fields]))) AND (("mortality"[Subheading] OR "mortality"[All Fields] OR "mortality"[MeSH Terms]) OR ("mortality"[Subheading] OR "mortality"[All Fields] OR "survival"[All Fields] OR "survival"[MeSH Terms]) OR ("metabolic diseases"[MeSH Terms] OR ("metabolic"[All Fields] AND "diseases"[All Fields]) OR "metabolic diseases"[All Fields]) OR ("diabetes mellitus"[MeSH Terms] OR ("diabetes"[All Fields] AND "mellitus"[All Fields]) OR "diabetes mellitus"[All Fields] OR "diabetes"[All Fields] OR "diabetes insipidus"[MeSH Terms] OR ("diabetes"[All Fields] AND "insipidus"[All Fields]) OR "diabetes insipidus"[All Fields]) OR ("alzheimer disease"[MeSH Terms] OR ("alzheimer"[All Fields] AND "disease"[All Fields]) OR "alzheimer disease"[All Fields] OR "alzheimer"[All Fields]))) AND "humans"[MeSH Terms]) AND (Review[ptyp] AND ("2006/01/01"[PDAT] : "2019/03/31"[PDAT]) AND "humans"[MeSH Terms]), Filters: Review Sort by Publication Date

**2) Search 2**

**Database** Snowball approach (articles from PubMed)

**Date** April 2019

**Total records** 481

**Strings**

**3) Search 3**

**Database** clinicaltrail.gov

**Date** 31^st^ March, 2019

**Total records** 262

**Strings** caloric restriction
