# Supplementary material for: Is Caloric Restriction Associated with Better Healthy Aging Outcomes? A Systematic Review and Meta-Analysis of Randomized Controlled Trials
Source: Nutrients. 2020 Jul 30;12(8):2290. doi: 10.3390/nu12082290 (PMC7468870; doi:10.3390/nu12082290)
Supplement: Supplementary file 1 [file nutrients-12-02290-s001.zip › Table S2 List of Included Studies.docx]

**Table S2 List of Included Studies**

**Armamento-Villareal 2012**

- Armamento-Villareal R, Sadler C, Napoli N, et al. Weight loss in obese older adults increases serum sclerostin and impairs hip geometry but both are prevented by exercise training. J Bone Miner Res. 2012;27(5):1215-1221. doi:10.1002/jbmr.1560

**Buchowski 2012**

- Buchowski MS, Hongu N, Acra S, Wang L, Warolin J, Roberts LJ. Effect of Modest Caloric Restriction on Oxidative Stress in Women, a Randomized Trial. PLoS One. 2012;7(10):1-10. doi:10.1371/journal.pone.0047079

**Haas 2014**

- Marilyn C. Haas. Calorie Restriction in Overweight SeniorS: Response of Older Adults to a Dieting Study: The CROSSROADS Randomized Controlled Clinical Trial. J Nutr Gerontol Geriatr. 2014;6(9):2166-2171. doi:10.1021/nl061786n.Core-Shell

**Heilbronn 2006**

- Heilbronn LK, de Jonge L, Frisard MI, DeLany JP, Larson Meyer DE, Rood J, Nguyen T, Martin CK, Volaufova J, Most MM, Greenway FL, Smith SR, Williamson DA, Deutsch WA, Ravussin E, the Pennington CALERIE Team: Effect of 6-month calorie restriction on biomarkers of longevity, metabolic adaptation, and oxidative stress in overweight subjects: a randomized controlled trial. 2006; JAMA 295:1539–1548 *****
- Larson-Meyer D, Heilbronn L, Redman L, et al. Effect of Calorie Restriction With or Without Exercise on Insulin Sensitivity, B-Cell Function, Fat Cell Size, and Extopic Lipid in Overweight Subjects. Nutr Metab Cardiovasc Dis. 2006;29(6):1337-1344. doi:10.2337/dc05-2565.Effect
- Civitarese AE, Carling S, Heilbronn LK, et al. Calorie restriction increases muscle mitochondrial biogenesis in healthy humans. PLoS Med. 2007;4(3):485-494. doi:10.1371/journal.pmed.0040076
- Redman LM, Heilbronn LK, Martin CK, Alfonso A, Steven R. Effect of calorie restriction with or without exercise on body composition and fat distribution. J Clin Endocrinol Metab. 2007;92(3):865-872. doi:10.1210/jc.2006-2184.Effect
- de Jonge L, Moreira EAM, Martin CK, Ravussin E. Impact of 6-month caloric restriction on autonomic nervous system activity in healthy, overweight, individuals. Obesity (Silver Spring). 2010;18(2):414-416. doi:10.1038/oby.2009.408
- Heilbronn LK, Jonge L De, Frisard MI, et al. Effect of 6-mo. calorie restriction on biomarkers of longevity, metabolic adaptation and oxidative stress in overweight subjects. Biomed Res. 2010;295(13):1539-1548. doi:10.1001/jama.295.13.1539.
- Lefevre M, Ph D, Redman LM, et al. Caloric restriction alone and with exercise improves CVD risk in healthy non-obese individuals. 2010;203(1):206-213. doi:10.1016/j.atherosclerosis.2008.05.036.CALORIC
- Lecoultre V, Ravussin E, Redman LM. The fall in leptin concentration is a major determinant of the metabolic adaptation induced by caloric restriction independently of the changes in leptin circadian rhythms. J Clin Endocrinol Metab. 2011;96(9):1512-1516. doi:10.1210/jc.2011-1286
- Redman LM, Veldhuis JD, Rood J, Smith SR, Williamson D, Ravussin E and PCT. The effect of caloric restriction interventions on growth hormone secretion in non-obese men and women. 2011;9(1):32-39. doi:10.1111/j.1474-9726.2009.00530.x.The
- Redman LM, Huffman KM, Landerman LR, et al. Effect of caloric restriction with and without exercise on metabolic intermediates in nonobese men and women. J Clin Endocrinol Metab. 2011;96(2):312-321. doi:10.1210/jc.2010-1971
- Tam CS, Frost EA, Xie W, Rood J, Ravussin E, Redman LM. No effect of caloric restriction on salivary cortisol levels in overweight men and women. Metabolism. 2014;63(2):194-198. doi:10.1016/j.metabol.2013.10.007
- Piacenza F, Malavolta M, Basso A., Costarelli L, Giacconi R, Ravussin E, Redman LM, Mocchegiani E. Effect of six-month caloric restriction on Cu bound to Ceruloplasmin in adult overweight subjects. 2015;26(8):876-882. doi:10.1016/j.jnutbio.2015.03.012

**Racette 2006**

- Racette SB, Weiss EP, Villareal DT, Arif H, Steger-May K, Schechtman KB, Fontana L, Klein S HJ. One Year of Caloric Restriction in Humans: Feasibility and Effects on Body Composition and Abdominal Adipose Tissue Susan. 2006;61(9):943-950 *****
- Weiss EP, Racette SB, Villareal DT, et al. Improvements in glucose tolerance and insulin action induced by increasing energy expenditure or decreasing energy intake: a randomized controlled trial. Am J Clin Nutr. 2006;84(5):1033-1042. doi:10.1093/ajcn/84.5.1033
- Fontana L, Villareal DT, Weiss EP, et al. Calorie restriction or exercise : effects on coronary heart disease risk factors . A randomized , controlled trial. 2007;63110:197-202. doi:10.1152/ajpendo.00102.2007.
- Weiss EP, Villareal DT, Racette SB, et al. Caloric Restriction But Not Exercise-Induced Reductions in Fat Mass Decrease Plasma Triiodothyronine Concentrations: A Randomized Controlled Trial. Rejuvenation Res. 2008;11(3):605-609. doi:10.1089/rej.2007.0622
- Fontana L, Weiss EP, Villareal DT, Klein S, Holloszy O. Long-term effects of calorie or protein restriction on serum IGF-1 and IGFBP-3 concentration in humans. Aging Cell. 2009;7(5):681-687. doi:10.1111/j.1474-9726.2008.00417.x

**Ravussin 2015**

- Ravussin E, Redman LM, Rochon J, et al. A 2-year randomized controlled trial of human caloric restriction: Feasibility and effects on predictors of health span and longevity. Journals Gerontol - Ser A Biol Sci Med Sci. 2015;70(9):1097-1104. doi:10.1093/gerona/glv057 *****
- Fontana L, Villareal DT, Das SK, et al. Effects of 2-year calorie restriction on circulating levels of IGF-1, IGF-binding proteins and cortisol in nonobese men and women: A randomized clinical trial. Aging Cell. 2016;15(1):22-27. doi:10.1111/acel.12400
- Meydani SN, Das SK, Pieper CF, et al. Long ‐ term moderate calorie restriction inhibits inflammation without impairing cell ‐ mediated immunity: a randomized controlled trial in non ‐ obese humans. 2016;8(7):1-16
- Villareal DT, Fontana L, Das SK, et al. Effect of Two-Year Caloric Restriction on Bone Metabolism and Bone Mineral Density in Non-Obese Younger Adults: A Randomized Clinical Trial. J Bone Miner Res. 2016;31(1):40-51. doi:10.1002/jbmr.2701
- Das S, Roberts S, Bhapkar M, et al. Body-composition changes in the Comprehensive Assessment of Long-term Effects of Reducing Intake of Energy (CALERIE)-2 study: a 2-y randomized controlled trial of calorie restriction in nonobese humans. Am J Clin Nutr. 2017;105(4):913-927
- Martin CK, Bhapkar M, Pittas AG, et al. Effect of Calorie Restriction on Mood, Quality of Life, Sleep, and Sexual Function in Healthy Nonobese Adults: The CALERIE 2 Randomized Clinical Trial. JAMA Intern Med. 2016;176(6):743-752. doi:10.1001/jamainternmed.2016.1189

**Sparks 2016**

- Sparks LM, Redman LM, Conley KE, et al. Effects of 12 Months of Caloric Restriction on Muscle Mitochondrial Function in Healthy Individuals. J Clin Endocrinol Metab. 2016;102(July):jc.2016-3211. doi:10.1210/jc.2016-3211

**Teng 2011**

- Teng NIMF, Shahar S, Manaf ZA, Das SK, Taha CSC, Ngah WZW. Efficacy of fasting calorie restriction on quality of life among aging men. Physiol Behav. 2011;104(5):1059-1064. doi:10.1016/j.physbeh.2011.07.007 *****
- Teng NIMF, Shahar S, Rajab NF, Manaf ZA, Johari MH, Ngah WZW. Improvement of metabolic parameters in healthy older adult men following a fasting calorie restriction intervention. Aging Male. 2013;16(4):177‐183. doi:10.3109/13685538.2013.832191
