# Supplementary material for: Is Caloric Restriction Associated with Better Healthy Aging Outcomes? A Systematic Review and Meta-Analysis of Randomized Controlled Trials
Source: Nutrients. 2020 Jul 30;12(8):2290. doi: 10.3390/nu12082290 (PMC7468870; doi:10.3390/nu12082290)
Supplement: Supplementary file 1 [file nutrients-12-02290-s001.zip › Table S3 Table of results.docx]

**Table S3 Table of results**

| Intervention description | Participants followed a balanced diet providing an energy deficit of 500–750 kcal/day from daily energy requirement. They met weekly with a dietitian for caloric intake adjustments. They were instructed to set weekly goals and to record their diet using food diaries. |
| --- | --- |
| Number in the Intervention Group | 26 |
| Control description | Participants did not receive advice to change their diet or activity habits. |
| Number in the Control Group | 27 |
| At risk/ ill | More than one factor for risk, mild-moderate frailty (impaired physical performance, oxygen consumption and/or light limitation in instrumental/basic activities of daily living). |
| Compliance / adherence to caloric restriction | High adherence rate to intervention. |
| Follow-up (months) | 12 |
| Outcomes (tools) | Body composition and bone mineral density (BMD) by dual energy X-ray absorptiometry (DXA)  Sclerostin (blood, immunoassay) |
| Results | Weight and lean body mass decreased significantly in the CR group (−9.6±1.2%; p<0.001; −3.2%±0.5%).  Serum sclerostin levels significantly and progressively increased at 6 months and at 12 months in the CR group (6.6±1.7% and 10.5±1.9%; p<0.05). Hip geometry parameters showed significant decreases in cross-sectional area, cortical thickness, and bone mineral density, and an increase in buckling ratio at the narrow neck, intertrochanteric and femoral shaft. Sclerostin levels were significantly associated with cortical thickness (r= −0.23, P=0.04) and with changes in BMD at the narrow neck (r= −0.22, P=0.05). |

**Armamento-Villareal 2012**

# **Buchowski 2012**

| Intervention description | Participants followed a diet contained approximately 75% of daily energy requirements. Participants received individualized diet and metabolic kitchen advices for consumption at home. Each diet consisted in daily portions divided into 3 meals and 3 snacks (52-62% of energy from carbohydrates, 25-29% from fat, 18-23% from protein, and 22-27 gr of fibers). In addition, multivitamin supplement was provided. They met weekly a dietitian to encourage compliance. |
| --- | --- |
| Number in the intervention group | 32 |
| Control description | Participants followed their habitual diet. |
| Number in the Control group | 8 |
| At risk/ ill | Overweight/obese participants. |
| Compliance / adherence to caloric restriction | Good |
| Follow-up (months) | 4 |
| Outcomes (tools) | Body Composition (DXA)  Blood Pressure (sphygmomanometer)  F2-Isoprostane (liquid chromatography/mass spectrometry)  Free fatty acids (spectrophotometry)  Hormones: Adiponectin, leptin (immunoassay, Luminex multiplexing technology)  Insulin (immunoassy)  Lipid Profile (blood, automated assay)  Inflammatory markers (immunoassay) |
| Results | One-month after the CR intervention, total body weight and body fat weight decreased in CR group more than in the control with differences of 2.7 kg and 1.1 kg, respectively. However, these differences were not statistically significant at the end of intervention (one-month follow-up) and 119-days after the baseline.  F2-Isoprostane decreased in CR group (P<0.001) by >50%. Both IL-8 and IL-12 were lower in CR subjects than controls after 1 month, while only IL-12 remained statistically lower than controls after 4 months. CR had no effect on systolic or diastolic blood pressure or circulating insulin, leptin, adiponectin, total cholesterol, LDL cholesterol, HDL cholesterol, free fatty acids, CRP, and triglycerides. |

# **Haas 2014**

| Intervention description | Participants partook in supervised exercise sessions and followed a reduced calorie diet: they received recommendation to emphasize ingestion of large quantities of high-bulk/low energy-density foods such as vegetables, fruits, high-fiber grains, and moderation in consumption of high energy-density foods such as meat, cheese, sugars, and fats; overall diet calories composition: 25% from protein, 47% from carbohydrate, and 28% from fat. |
| --- | --- |
| Number in Intervention Group | 55 |
| Control description | Participants participated in supervised exercise sessions. |
| Number in the Control group | 54 |
| At risk/ ill | More than one factor for risk for health and comorbid conditions affected by high weight status (obese subjects). |
| Compliance / adherence to caloric restriction | Not reported. |
| Follow-up (months) | 12 |
| Outcomes (tools) | Body Composition (magnetic resonance imaging)  Fasting Glucose (blood, automated assay) |
| Results | Visceral Adipose Tissue decreased in both groups (-192.6 cm^3^ in CR and -21.9 cm^3^ in controls)  Blood Glucose was better in CR than Control (-7.2 mg/dL in CR; 1.2 in controls) |

# **Heilbronn 2006**

| Intervention description | Participants followed a reduced-calorie diet (-25%) and were provided with all their food at baseline and for the first 12 weeks after randomization. Participants ate 2 meals at the study centre each week-day with 1 meal plus snacks packaged for take-out; then, they self-selected their diet based on individual calorie targets (weeks 13-22); finally, they returned to the in-feeding protocol (weeks 22-24). |
| --- | --- |
| Number in the intervention group | 12 |
| Control description | Participants continued their diet. |
| Number in the Control group | 11 |
| At risk/ ill | Overweight/obese participants. |
| Compliance / adherence to caloric restriction | Good: high level of compliance to intervention of participants |
| Follow-up (months) | 6 |
| Outcomes (tools) | Autonomic Nervous System (spectral analysis of heart rate variability)  Blood pressure (sphygmomanometer)  Body composition (DXA)  Body temperature and total daily energy expenditure (TDEE) (not reported)  Bone mass and fasting serum bone markers (DXA, blood immunoassay)  Copper bound to ceruloplasmin (high-performance liquid chromatography/mass spectrometry)  DHEAS (blood, immunoassay)  Fasting insulin and insulin sensitivity (immunoassay, intravenous glucose tolerance test)  GH, ghrelin and IGF-1 (blood, immunoassay)  Lipid profile (automated assay)  Mitochondrial Content in Skeletal Muscle (qRT-PCR, Mitotracker Green probe (Molecular Probes))  Nitric Oxide and Mitochondrial Biogenesis (qRT-PCR)  Protein carbonyls (modified 2,4-dinitrophenylhydrazine (DNPH assay)  Salivar Cortisol (immunoassay)  Thyroid Hormones (T3) (immunoassay)  Urinary catecholamines (not reported) |
| Results | Hearth Rate decreased not significantly in CR at 6 months. SNS activity not changed in CR group at 6 months.  No significant change was seen for blood pressure.  CR significantly reduced body weight (−8±1 kg, 10±1%), fat mass (-24±3%), visceral adipose tissue (-28±4%; p<0.005), subcutaneous abdominal adipose tissue (-26±4%; p<0.005), deep subcutaneous abdominal adipose (-29±5%; p<0.005), fat cell mass (-19±4%; p<0.001), intrahepatic lipid (-37±10%; p<0.01). No change was seen for intramyocellular lipid.  CR decreased TDEE (-135±42 kcal/d) and 24-h core body temperature (-0,2 C°; p<0.05).  There was no change for bone mineral density for total body or hip in the CR group. Bone alkaline phosphatase was reduced by 16% (-23±10%) in the CR group; no change in cross-linked N-telopeptide and osteocalcin was seen.  Copper bound to ceruloplasmin did not change. No change in DHEAS was seen in the CR group at 6 months.  Fasting insulin significantly decreased in CR group (p<0.01); there was a mild improvement after CR in insulin sensitivity (40±20%, P=0.08) and acute insulin response to glucose (29±7%). The improvement of insulin sensitivity was related to the reduction in percent fat (r=−0.46, P = 0.002), visceral adipose tissue (r=−0.51, P<0.01), subcutaneous abdominal adipose tissue (r=−0.32, P<0.05), and deep subcutaneous abdominal adipose tissue (r =−0.47, P<0.01).  There was no change in GH or IGF1 concentrations for CR group. Ghrelin concentration increased significantly in CR group at 6 months (7±1%; p<0.03).  Leptin significantly decreased in CR (p<0.001). The change in leptin mesor was significantly related to fat mass (p<0.04).  Triacylglycerol decreased in CR group at 3 and 6 months (-31 ± 15 mg/dL, p<0.05). LDL cholesterol did not change at 6 months in CR group, HDL did not change at 3 months but increased at 6 months.  CR increased the expression levels of TFAM and PPARGC1A suggesting an induction of mitochondrial biogenesis. There was also an induction of mtDNA content (marker of mitochondrial mass, 35%±5%; p=0.005). SIRT1 mRNA is increased in the CR (113±24%; p=0.016).  Endothelial nitric oxide synthase was increased by 67% (p=0.002). Presenilin-associated, rhomboid-like protein (PARL; p=0.008) and AMP-activated protein kinase alpha 2 (AMPK-a2) mRNA (p=0.03) increased relative to baseline in the CR group. No change for mitochondrial enzymes of the TCA (tricarboxylic acid) cycle (citrate synthase), beta-oxidation (beta-hydroxyacyl-CoA dehydrogenase), and electron transport chain was observed. There was no change in protein carbonyls in the CR group, but DNA damage are reduced at 6 month (-0,56 AU, p<0.005).  No change was recorded for salivary cortisol. T3 was reduced by CR at 3 (-10.2 ng/dL; p<0.01) and 6 months (-8.9ng/dL; p<0.02), similar result were seen for T4 (p<0.05). Urinary epinephrine excretion did not change in the CR group, while norepinephrine decreased (p=0.05). |

# **Racette 2006**

| Intervention description | Participants received decreased (-20%) energy intake from baseline without a change in energy expenditure; after 4-weeks and after 3-months, participants received meals from the General Clinical Research Center metabolic kitchen for 5 consecutive days to educate them on the serving sizes; they weekly met dietitian for support during the first 6 months, and then less frequently. In addition, they were instructed to set weekly goals and were encouraged to use food diaries. Supplement of multivitamin with minerals was added. |
| --- | --- |
| Number in the intervention group | 18 |
| Control description | Healthy lifestyle without caloric restriction |
| Number in the Control group | 10 |
| At risk/ ill | Overweight/obese participants. |
| Compliance / adherence to caloric restriction | Energy intake decreased significantly in CR group during the first 6 months, then adherence was lower during the following months. Globally, the CR group reduced caloric intake about 11.5% (±2.1) |
| Follow-up (months) | 12 |
| Outcomes (tools) | Adiponectin (blood, immunoassay)  Blood pressure and CRP (oscillometric BP monitor, ELISA)  Body composition (DXA)  Cortisol and free fatty acids (blood, immunoassay)  IGFBP-3 and IGF-1(blood, immunoassay)  Insulin and insulin sensitivity (blood, immunoassay)  Lipid profile (automated assay)  Thyroid hormones T_4 ,_ T_3_ (fluorescence, immunoassay)  TNFα (blood, immunoassay) |
| Results | Serum adiponectin increased significantly in CR group (p=0.07), TNFα did not change; TNFα/adiponectin ratio decreased significantly (p<0.05). CRP and systolic and diastolic blood pressure decreased in CR group, but not significantly.  CR group lost about 10.7% of baseline weights after 12 months that mean −8.0 ± 0.9 kg (p=0.0005). Also, reduction in whole body fat mass was significant (p=0.0001), about 77% of total weight lost. Visceral (p=0.05) and subcutaneous abdominal adipose tissue (p=0.02) decreased significantly in the CR.  Cortisol and free fatty acids changed after intervention in CR group more than in control (CR -4.0±4.1 vs. control -2.2±4.2 for cortisol; CR -68±306 vs. control 13±152 for free fatty acids), but reductions were not statistically significant respect to control. There were not changes in serum IGF-1 concentration or IGF-1/IGFBP-3 ratio in CR group.  Insulin sensitivity increased significantly in CR group (p<0.005) with respect to controls, consequently glucose (p=0.03) and insulin AUC (p<0.05) decreased. HOMA-IR significantly decreased (p<0.05).  Total cholesterol decreased significantly in CR group (p<0.05). LDL decreased in CR group (p<0.05) but HDL not increased; Total cholesterol/HDL ratio decreased significantly (p<0.05). No change for triglycerides was seen.  Plasma concentrations of T3 decreased significantly in CR (-9.8 ±2.0 ng/dL; p <0.0001) group. No changes for T4, TSH and FT4 were seen. |

# **Ravussin 2015**

| Intervention description | Participants followed a diet with 25% fewer calories than calculated at baseline. For the first 27 days, they were fed their assigned caloric prescription (3 different provided 9-day diets). The food provision was used to educate them on portion size, energy content and different types of dietary patterns. They received a structured curriculum and manual-driven counseling in group and individual sessions (by both clinical psychologists and nutritionists), with continuous feedback and communication between the participants and the interventionists. |
| --- | --- |
| Number in the intervention group | 143 |
| Control description | Participants continued their current diet. |
| Number in the Control group | 75 |
| At risk/ ill | Apparently healthy people. |
| Compliance / adherence to caloric restriction | In CR group, energy intake was reduced by 19.5% (±0.8) during the first 6 months, and by 9.1% (±0.7) below baseline for the remaining 18 months. In control group, energy intake was not changed. |
| Follow-up (months) | 12 and 24 |
| Outcomes (tools) | Blood pressure (not reported)  Body composition (DXA)  Bone turnover and related hormones (DXA, immunoassay)  Cortisol and IGF-1 (immunoassay)  Insulin (automated assay)  Leptin (immunoassay)  Lipid profile (automated assay)  Markers of inflammation (immunoassay)  Sex hormones (not reported)  TDEE  Resting Metabolic Rate (RMR) and Core Temperature (not reported)  Thyroid hormones (not reported)  Quality of Life (QoL): Rand 36-Item Short Form (SF-36), with higher scores indicate better QoL, and Perceived Stress Scale (PSS), with higher scores indicate higher levels of perceived stress.  Mood indicators: Beck Depression Inventory II (BDI-II) and Profile of Mood States (PMS); higher scores indicate worse mood in both scales.  Sleep quality: Pittsburgh Sleep Quality Index (PSQI), higher scores indicate worse sleep quality.  Sexual function: Derogatis Interview for Sexual Function–Self-report (DISFS), higher scores indicate better functioning. |
| Results | Systolic and diastolic pressure were greater in CR than in AL (p=0.001) as well as glucose control.  The CR group lost 7.6±0.32 kg (p<0.001 at 24 months). CR group decreased also fat mass and fat-free mass (p<0.01 for both). Fat-free mass decreased more in men.  BMD decreased significantly in CR group at the lumbar spine (−0.013±0.003 vs. 0.007±0.004 g/cm2; p<0.001), total hip (−0.017±0.002 vs. 0.001±0.003 g/cm2; p<0.001), femoral neck (−0.015±0.003 vs. −0.005±0.004 g/cm2; p=0.03) and other regional hip sites at 12 months without differences between males and females. Bone resorption markers like C-terminal telopeptide of type I collagen (0.098±0.012 vs. 0.025±0.015 μg/L; p<0.001) and tartrate-resistant acid phosphatase isoform (0.4±0.1 vs. 0.2±0.1 U/L; p=0.004) significantly increased at 6 months and remained elevated at 12 months in the CR group. Bone formation markers like bone specific alkaline phosphatase and intact N-terminal propeptide of type I remained stable at 6 months; bone specific alkaline phosphatase decreased at 12 months (−1.4±0.4 vs. −0.3±0.5 U/L; p=0.047). 25ODH increased in the CR group but not accompanied by change in PTH.  Cortisol increased significantly in the CR group at 12 months (p=0.003). No changes were observed for IGF-1, while CR increased serum levels of IGBP-1(by 25.2% and 21% after 1 and 2 years of CR) resulting in a decreased IGF-1/IGFBP-1 ratio (-42% in CR group).  Leptin decreased significantly in the CR group compared to control both at month 12 and 24 (p<0.0001).  Triglycerides and total and LDL cholesterol decreased significantly, while HDL increased significantly after CR.  Insulin decreased in CR group at 12 and 24-months.  CR decreased CRP significantly both months 12 and 24 (p=0.001). TNF-alpha decreased significantly in the CR group at month 24 (p<0.0001). Serum concentrations of CRP and TNF-α were 40% and 50% lower in the CR. Physical activity significantly decreased in the CR group, but not grip strength.  Sex-hormone binding globulin (SHBG) improved in men after CR at month 12 (BGD, 0.76; 95% CI, 0.40–1.12 μg/mL. Instead free testosterone levels decreased in the CR group at month 12 (BGD, −2.98; 95% CI, −4.75 to −1.21 ng/dL). No significant changes were observed for luteinizing hormone, follicle-stimulating hormone and total testosterone.  TDEE decreased in CR group compared to AL at 12-months (44 ± 26 kcal/d; p <0 .001). RMR decreased significantly in CR group compared to AL group at 12-months (48 ± 9 vs 14 ± 12 kcal/d in AL, p = 0.04). The core temperature decreased slightly but not significantly compared to AL group.  Thyroid Hormones decreased significantly at 12-months (16 ± 1.5%).  Secondary outcomes:  After 24-months follow-up there was little improvement on mood measured by BDI-II (MD -0.76 95% CI -1.41 to -0.11) and on PMS domain related to tension (MD -0.79 95% CI -1.38 to -0.19). No effect was recorded on the other five domains of PMS, as well as on its total score.  QoL did not show any difference between intervention and control, except on general health domain of SF-36 (MD 6.33, 95% CI 3.68 to 8.98; and 6.45, 95% CI 3.93 to 8.98, at 12 and 24-months follow-ups, respectively). No effect was recorded on the PSS during both follow-ups.  After 12-months follow-up there was a slightly improvement on the PSQI scale related to sleep duration domain (MD -0.26 95% CI -0.49 to -0.02), but not on the subjective sleep quality and total score of the scale.  No effect was recorded on DISFS in relation to sexual function. |

# **Sparks 2016**

| Intervention description | Participants were assigned a reduction of energy intake by 25%: provision of meals for the first 27 days and regular group and individual meetings with trained interventionists. |
| --- | --- |
| Number in the intervention group | 33 |
| Control description | Maintain habitual energy intake on ad libitum |
| Number in the Control group | 18 |
| At risk/ ill | More than one factor for risk |
| Compliance / adherence to caloric restriction | Only participants who complied with the intervention protocols and completed the intervention were included in analysis: only one participant was excluded. |
| Follow-up (months) | 12 |
| Outcomes (tools) | Blood pressure (not reported)  Body composition (DXA)  Insulin and lipid profile (blood, automated assay)  Muscle lipid content and Muscle Mitochondrial Function and Transcriptional Profiles (H-MRS using the PRESS technique) |
| Results | Diastolic blood pressure (p=0.04), insulin (p=0.02), total cholesterol (p=0.02), and LDL decreased in CR group at 12 months but not significantly in Control.  Weight, BMI, fat mass, total % body fat, total lean body mass, and leg lean body mass decreased significantly in CR group (p=0.001 for all).  Intramyocellular lipid was lower after CR (p=0.04). Muscle ATP synthesis, resting ATPflux, O2 uptake, and P/O did not differ significantly. SIRT1 is increased in CR group (p = 0.03) whereas mtDNA copy number decreased in CR (p=0.003). There was also a down regulation of genes related to NO in CR group.  Fasting insulin decreased in CR group and difference with control is statistically significant (from 5.1 mIU/mL ±2.4 to 4.0 mIU/mL ± 2.0 in CR; from 6.5 mIU/mL ±2.4 to 6.5 mIU/mL ±2.5 in control; p<0.001). |

# **Teng 2011**

| Intervention description | Participants were assigned a reduction of 300 to 500 kcal/day from their habitual energy intake combined with two days of Muslim Sunnah fasting per week; they received individual and group counselling. |
| --- | --- |
| Number in the intervention group | 14 |
| Control description | Maintain their lifestyle. |
| Number in the Control group | 14 |
| At risk/ ill | More than one factor for risk and diagnosed hypertension. |
| Compliance / adherence to caloric restriction | Good: energy intake changed from baseline (1681±191 kcal/day) to 3 month-follow-up (1371±186 kcal/day). All participants followed the CR regimen. |
| Follow-up (months) | 3 |
| Outcomes (tools) | Body Composition (bioelectrical impedance analysis)  Quality Of life (Quality of Life (QoL): Rand 36-Item Short Form (SF-36), with higher scores indicate better QoL, and Perceived Stress Scale (PSS), with higher scores indicate higher levels of perceived stress.  Mood indicators: Beck Depression Inventory II (BDI-II); higher scores indicate worse mood in both scales  Sleep quality: Pittsburgh Sleep Quality Index (PSQI), higher scores indicate worse sleep quality. |
| Results | There was a significant reduction in body weight at 3-months (69.3±6.0 CR; 73.7±8.4 Control; p<0.001), BMI (26.3±1.9 CR; 26.8±1.9 Control; p<0.001) and fat mass (52.2±4.1 CR; 54.8±5.2 Control; p<0.05).  The CR determined a greater increase in vitality domain of SF-36 (8.7% FCR vs 5.9% in the control group; p<0.05), the physical health component of SF-36 (8.3% in the FCR group as compared to 6.2% in the control group; p<0.05). No significant change was seen for PSS, as well as for the other two scales measuring mood and sleep quality, respectively. |

**Table 1.** Meta-analysis comparing CR regimens with control: MD or SMD, 95%CI and I-squared percentages for Weight, Fat mass, BMI, SBP, DBP, HDL, LDL, Total cholesterol, Fasting blood glucose, Fasting blood insulin

| **Outcome** | **By subgroup meta-analysis** | | **No. of studies** | **MD (95% CI)** | **I2 (%)** |
| --- | --- | --- | --- | --- | --- |
| **Weight ∞** | Overall |  | 6 | -7.90 (-7.99, -7.81) | 0.0 |
|  |  | Normal weight | 2 | -7.90 (-7.99, -7.81) | 0.0 |
|  | By BMI | Overweight | 3 | -6.50 (-10.61, -2.40) | 0.0 |
|  |  | Obese | 1 | -3.30 (-17.72, 11.12) | - |
|  | By follow-up | ≤5 months from baseline | 2 | -4.26 (-9.33, -0.80) | 0.0 |
|  |  | ≥6 ≤ 11 months from the baseline | 1 | -8.70 (-17.36, -0.04) | - |
|  |  | ≥12 months from the baseline | 3 | -7.90 (-7.99, -7.81) | 0.0 |
| **Fat mass ∞** | Overall |  | 5 | -4.40 (-6.69, -0.45) | 85.7 |
|  | By follow-up | ≤6 months from baseline | 2 | -1.91 (-3.37, -0.45) | 0.0 |
|  |  | >6 months from baseline | 3 | -5.80 (-5.87, -5.72) | 0,0 |
|  | By BMI | Normal weight | 2 | -5.80 (-5.87, -5.72) | 0.0 |
|  |  | Overweight | 2 | -3.64 (-7.70, -0.41) | 76.5 |
|  |  | Obese | 1 | -2.40 (-12.72, 7.92) | - |
| **BMI **** | Overall |  | 5 | -2.68 (-3.51, -1.86) | 69.6 |
|  | By follow-up | Follow up ≤ 5 months from baseline | 1 | -0.50 (-1.91, 0.91) | - |
|  |  | Follow up ≥6 ≤ 11 months from the baseline | 1 | -4.30 (-6.18, -2.42) | - |
|  |  | Follow up≥12 months from the baseline | 3 | -2.70 (-2.73, -2.67) | 0.0 |
| **SBP ◊** | Overall |  | 4 | -2.45 (-5.24, 0.35) | 77.9 |
| **DBP ◊** | Overall |  | 4 | -0.65 (-2.03, 0.72) | 46.2 |
| **LDL cholesterol +** | Overall |  | 3 | -22.03 (-29.11, -14.96) | 39.7 |
| **HDL cholesterol +** | Overall |  | 3 | -0.04 (-3.14, 3.06) | 46.2 |
| **Total cholesterol +** | Overall |  | 3 | -12.72 (-23.77, -1.77) | 60.9 |
| **Fasting insulin *** | Overall |  | 5 | -2.76 (-4.42, -1.10) | 94.3 |
| **Fasting glucose +** | Overall |  | 3 | -1.31 (-2.38, -0.24) | 0.0 |

* MD is expressed in mIU/L; ** MDs are expressed in kg/m^2^; ∞ MDs are expressed in kg; ◊ MDs are expressed in mmHg; + MDs are expressed in mg/dl.

**Table 4.** Sensitivity analysis: meta-analysis by balanced and non-balanced groups at baseline for SBP, DBP, LDL, HDL, Total cholesterol, Fasting blood insulin, Fasting blood glucose

| **Outcome** | **By subgroup meta-analysis** | **No. of studies** | **MD (95% CI)** | **I2** |
| --- | --- | --- | --- | --- |
| **SBP ◊** | balanced groups | 2 | -0.92 (-2.13, 0.29) | 0.0% |
| **SBP ◊** | non-balanced groups | 2 | -4.46 (-5.96, -2.96) | 0.0% |
| **DBP ◊** | balanced groups | 2 | 0.11 (-0.97. 1.19) | 0.0% |
| **DBP ◊** | non-balanced groups | 2 | -1.51 (-2.69, -0.34) | 0.0% |
| **LDL cholesterol +** | balanced groups | 2 | -19.63 (-30.95, -8.31) | 66.5% |
| **LDL cholesterol +** | non-balanced groups | 1 | -27.07 (-40.91, -13.23) | .% |
| **HDL cholesterol +** | balanced groups | 2 | -1.56 (-3.72, 0.59) | 0.0% |
| **HDL cholesterol +** | non-balanced groups | 1 | 2.71 (-1.78, 7.19) | .% |
| **Total cholesterol +** | balanced groups | 2 | -6.98 (-12.84, -1.11) | 0.0% |
| **Total cholesterol +** | non-balanced groups | 1 | -24.36 (-39.29, -9.43) | .% |
| **Fasting insulin *** | balanced groups | 3 | -3.11 (-6.44, 0.22) | 95.0% |
| **Fasting insulin *** | non-balanced groups | 2 | -2.13 (-4.70, 0.44) | 72.2% |
| **Fasting glucose +** | balanced groups | 2 | -1.24 (-1.42, -0.06) | 8.0% |
| **Fasting glucose +** | non-balanced groups | 1 | -3.40 (-8.37, 1.57) | 0.0% |

* MDs are expressed in mIU/L; ◊ MDs are expressed in mmHg; + MDs are expressed in mg/dl.
